# Vedolizumab for induction and maintenance of remission in Crohn's disease

**DOI:** 10.1002/14651858.CD013611.pub2

**Published:** 2023-07-17

**Authors:** Samuel Hui, Vassiliki Sinopoulou, Morris Gordon, Ghazaleh Aali, Anuj Krishna, Nik Sheng Ding, Ray K Boyapati

**Affiliations:** Department of Gastroenterology, Monash Medical CentreMelbourneAustralia; School of MedicineUniversity of Central LancashirePrestonUK; Institute for Health Informatics ResearchUniversity College LondonLondonUK; Austin HealthMelbourneAustralia; Department of GastroenterologySt Vincent's HospitalMelbourneAustralia; Department of GastroenterologyMonash Medical CentreMelbourneAustralia

**Keywords:** Adult, Humans, Antibodies, Monoclonal, Humanized, Antibodies, Monoclonal, Humanized/adverse effects, Colitis, Ulcerative, Colitis, Ulcerative/drug therapy, Crohn Disease, Crohn Disease/drug therapy, Crohn Disease/surgery, Inflammatory Bowel Diseases, Inflammatory Bowel Diseases/drug therapy, Remission Induction

## Abstract

**Background:**

Vedolizumab blocks inflammatory activity within the gastrointestinal tract. Systematic reviews have demonstrated the efficacy of vedolizumab in ulcerative colitis and inflammatory bowel disease in general. This systematic review and meta‐analysis summarises the current evidence of vedolizumab in the induction and maintenance of remission in Crohn's disease.

**Objectives:**

To evaluate the benefits and harms of vedolizumab versus placebo for the induction and maintenance of remission in people with Crohn's disease.

**Search methods:**

We used standard, extensive Cochrane search methods. The latest search date was 30 November 2022.

**Selection criteria:**

We included randomised controlled trials (RCTs) and quasi‐RCTs comparing vedolizumab to placebo for the induction or maintenance of remission in people with Crohn's disease.

**Data collection and analysis:**

We used standard Cochrane methods. For induction studies, the primary outcome was 1. clinical remission, and secondary outcomes were rates of 2. clinical response, 3. adverse events, 4. serious adverse events, 5. surgery, 6. endoscopic remission and 7. endoscopic response. For maintenance studies, the primary outcome was 1. maintenance of clinical remission, and secondary outcomes were rates of 2. adverse events, 3. serious adverse events, 4. surgery, 5. endoscopic remission and 6. endoscopic response. We used GRADE to assess certainty of evidence.

**Main results:**

We analysed induction (4 trials, 1126 participants) and maintenance (3 trials, 894 participants) studies representing people across North America, Europe, Asia and Australasia separately. One maintenance trial administered subcutaneous vedolizumab whilst the other studies used the intravenous form. The mean age ranged between 32.6 and 38.6 years.

Vedolizumab was superior to placebo for the induction of clinical remission (71 more per 1000 with clinical remission with vedolizumab; risk ratio (RR) 1.61, 95% confidence interval (CI) 1.20 to 2.17; number needed to treat for an additional beneficial outcome (NNTB) 13; 4 studies; high‐certainty evidence) and superior to placebo for inducing clinical response (105 more per 1000 with clinical response with vedolizumab; RR 1.43, 95% CI 1.19 to 1.71; NNTB 8; 4 studies; high‐certainty evidence). For the induction phase, vedolizumab may be equivalent to placebo for the development of serious adverse events (9 fewer serious adverse events per 1000 with vedolizumab; RR 0.91, 95% CI 0.62 to 1.33; 4 studies; low‐certainty evidence) and probably equivalent to placebo for overall adverse events (6 fewer adverse events per 1000 with vedolizumab; RR 1.01, 95% CI 0.93 to 1.11; 4 studies; moderate‐certainty evidence).

Vedolizumab was superior to placebo for the maintenance of clinical remission (141 more per 1000 with maintenance of clinical remission with vedolizumab; RR 1.52, 95% CI 1.24 to 1.87; NNTB 7; 3 studies; high‐certainty evidence). During the maintenance phase, vedolizumab may be equivalent to placebo for the development of serious adverse events (3 fewer serious adverse events per 1000 with vedolizumab; RR 0.98, 95% CI 0.68 to 1.39; 3 studies; low‐certainty evidence) and probably equivalent to placebo for the development of overall adverse events (0 difference in adverse events per 1000; RR 1.00, 95% CI 0.94 to 1.07; 3 studies; moderate‐certainty evidence).

**Authors' conclusions:**

High‐certainty data across four induction and three maintenance trials demonstrate that vedolizumab is superior to placebo in the induction and maintenance of remission in Crohn's disease. Overall adverse events are probably similar and serious adverse events may be similar between vedolizumab and placebo during both induction and maintenance phases of treatment. Head‐to‐head research comparing the efficacy and safety of vedolizumab to other biological therapies is required.

## Plain language summary

**Vedolizumab for induction and maintenance of remission in Crohn's disease**

**Key messages**

– Vedolizumab is effective in inducing and maintaining remission in people with Crohn's disease when compared to placebo (a dummy treatment).

– People receiving vedolizumab are probably no more likely than people receiving placebo to experience side effects, and may be no more likely to experience serious side effects, during induction or maintenance treatment.

**What did we want to find out?**

Crohn's disease is a chronic inflammatory condition affects the gastrointestinal tract (the gut). Vedolizumab is called a 'biological' medication that blocks an important protein called α4β7 that is involved in gut inflammation and results in gut‐selective anti‐inflammatory activity. We wanted to find out whether vedolizumab is effective and safe in treating Crohn's disease.

**What did we do?**

We searched for studies that compared vedolizumab to placebo in people with Crohn's disease. We compared and summarised their results, and rated our confidence in the evidence, based on factors such as study methods and sizes.

**What did we find?**

We found four studies including 1126 participants that investigated vedolizumab's effect in inducing remission (where the disease is no longer active), and three studies including 894 participants that investigated its effect in maintaining remission.

Compared to placebo, vedolizumab was more effective at inducing remission (4 studies in 1126 participants) and maintaining remission (3 studies in 894 participants) in Crohn's disease. Vedolizumab is more effective than placebo for inducing a clinical response (i.e. disease entering remission; 4 studies in 1126 participants). It is probably as likely as placebo to cause overall side effects and as likely as placebo to cause serious side effects during both induction (4 studies in 1126 participants) or maintenance treatment (3 studies in 894 participants).

**What are the limitations of the evidence**

We are highly certain about the evidence for induction and maintenance of remission. However, there were limitations in comparing vedolizumab to placebo for serious and overall side effects due to our low confidence in the results.

**How up‐to‐date is this evidence?**

The evidence is up to date to November 2022.

## Summary of findings

**Summary of findings 1:** Vedolizumab compared to placebo for induction of remission in Crohn's disease

**Vedolizumab compared to placebo for induction of remission in Crohn's disease**						
**Patient or population:** children or adults with Crohn's disease **Setting:** inpatient or outpatient **Intervention:** vedolizumab (induction therapy) **Comparison:** placebo						
**Outcomes**	Anticipated absolute effects^*^ (95% CI)		**Relative effect (95% CI)**	**№ of participants (studies)**	**Certainty of the evidence (GRADE)**	**Comments**
	**Risk with placebo**	**Risk with vedolizumab**				
**Induction of clinical remission (6–10 weeks)**	**Study population**		**RR 1.61** (95% CI 1.20 to 2.17)	1126 (4 studies)	**High**^a^ ⊕⊕⊕⊕	Outcome occurred in 19.8% in the vedolizumab group compared with 11.6% in the placebo group; the NNTB was 13.
	116 per 1000	**71 more per 1000** (23 more to 136 more)				
**Induction of clinical response (6–10 weeks)**	**Study population**		**RR 1.43** (95% CI 1.19 to 1.71)	1126 (4 studies)	**High**^a^ ⊕⊕⊕⊕	Outcome occurred in 36.9% in the vedolizumab group compared with 24.2% in the placebo group; the NNTB was 8.
	242 per 1000	**105 more per 1000** (46 more to 172 more)				
**Adverse events (6–10 weeks)**	**Study population**		**RR 1.01** (95% CI 0.93 to 1.11)	1126 (4 studies)	**Moderate**^a,b^ ⊕⊕⊕⊝	Outcome occurred in 6.4% in the vedolizumab group compared with 6.2% in the placebo group.
	619 per 1000	**6 fewer per 1000** (43 fewer to 68 more)				
**Serious adverse events (6–10 weeks)**	**Study population**		**RR 0.91** (95% CI 0.62 to 1.33)	1126 (4 studies)	**Low**^a,c^ ⊕⊕⊝⊝	Outcome occurred in 9% in the vedolizumab group compared with 9.2% in the placebo group.
	92 per 1000	**9 fewer per 1000** (35 fewer to 30 more)				
*The risk in the intervention group (and its 95% confidence interval) is based on the assumed risk in the comparison group and the **relative effect** of the intervention (and its 95% CI). **CI:** confidence interval; **NNTB:** number needed to treat for an additional beneficial outcome; **RR:** risk ratio.						
**GRADE Working Group grades of evidence** **High certainty:** we are very confident that the true effect lies close to that of the estimate of the effect. **Moderate certainty:** we are moderately confident in the effect estimate: the true effect is likely to be close to the estimate of the effect, but there is a possibility that it is substantially different. **Low certainty:** our confidence in the effect estimate is limited: the true effect may be substantially different from the estimate of the effect. **Very low certainty:** we have very little confidence in the effect estimate: the true effect is likely to be substantially different from the estimate of effect.						

^a^Not downgraded for study limitations even though there were some risk of bias domains that were unclear. Overall these were not considered serious. ^b^Downgraded once for imprecision due to a moderately narrow confidence interval. ^c^Downgraded twice for imprecision due to a wide confidence interval.

**Summary of findings 2:** Vedolizumab compared to placebo for maintenance of remission in Crohn's disease

**Vedolizumab compared to placebo for maintenance of remission in Crohn's disease**						
**Patient or population:** children or adults with Crohn's disease **Setting:** inpatient or outpatient **Intervention:** vedolizumab (maintenance therapy) **Comparison:** placebo						
**Outcomes**	Anticipated absolute effects^*^ (95% CI)		**Relative effect (95% CI)**	**№ of participants (studies)**	**Certainty of the evidence (GRADE)**	**Comments**
	**Risk with Placebo**	**Risk with vedolizumab**				
**Maintenance of clinical remission (52–60 weeks)**	**Study population**		**RR 1.52** (95% CI 1.24 to 1.87)	894 (3 studies)	**High**^a^ ⊕⊕⊕⊕	Outcome occurred in 42.5% in the vedolizumab group compared with 27.1% in the placebo group; the NNTB was 7.
	271 per 1000	**141 more per 1000** (65 more to 236 more)				
**Adverse events (52–60 weeks)**	**Study population**		**RR 1.00** (95% CI 0.94 to 1.07)	894 (3 studies)	**Moderate**^b^ ⊕⊕⊕⊝	Outcome occurred in 80% in the vedolizumab group compared with 80.3% in the placebo group.
	803 per 1000	**0 difference per 1000** (48 fewer to 56 more)				
**Serious adverse events (52–60 weeks)**	**Study population**		**RR 0.98** (95% CI 0.68 to 1.39)	894 (3 studies)	**Low**^c^ ⊕⊕⊝⊝	Outcome occurred in 13.1% in the vedolizumab group compared with 13.7% in the placebo group.
	137 per 1000	**3 fewer per 1000** (44 fewer to 54 more)				
*The risk in the intervention group (and its 95% confidence interval) is based on the assumed risk in the comparison group and the **relative effect** of the intervention (and its 95% CI). **CI:** confidence interval; **NNTB:** number needed to treat for an additional beneficial outcome; **RR:** risk ratio.						
**GRADE Working Group grades of evidence** **High certainty:** we are very confident that the true effect lies close to that of the estimate of the effect. **Moderate certainty:** we are moderately confident in the effect estimate: the true effect is likely to be close to the estimate of the effect, but there is a possibility that it is substantially different. **Low certainty:** our confidence in the effect estimate is limited: the true effect may be substantially different from the estimate of the effect. **Very low certainty:** we have very little confidence in the effect estimate: the true effect is likely to be substantially different from the estimate of effect.						

^a^Not downgraded for study limitations even though there were some risk of bias domains that were unclear. Overall these were not considered serious. ^b^Downgraded once for imprecision due to a moderately narrow confidence interval. ^c^Downgraded twice for imprecision due to a wide confidence interval.

## Background

### Description of the condition

Crohn's disease (CD) is a chronic inflammatory bowel disease (IBD) characterised by transmural inflammation of the gastrointestinal tract. Its pathophysiology is thought to involve a complex interplay between genetic susceptibility, immune and environmental factors (Boyapati 2015). Worldwide, the incidence of CD is increasing, with the highest incidence in westernised nations (Molodecky 2012).

Symptoms depend on the area of bowel involved but often include diarrhoea, abdominal pain, gastrointestinal bleeding and weight loss. Complications may further arise with the development of stricturing or fistulising disease. Conventional therapy is with corticosteroids followed by an immunomodulator (methotrexate, azathioprine, 6‐mercaptopurine) or a tumour necrosis factor (TNF) inhibitor (infliximab, adalimumab, certolizumab). However, this approach results in up to 55% to 60% of people failing to achieve remission at one year following diagnosis (D'Haens 2008). Despite the advent of TNF inhibitors, many people have either primary non‐response or secondary loss of response and, as such, new therapies with different mechanisms have been advanced.

### Description of the intervention

Vedolizumab is a humanised monoclonal antibody which inhibits the α4β7 integrin. Integrins are adhesion molecules which allow for lymphocyte trafficking, an important process in T‐cell‐mediated inflammation. The value of inhibiting α4 integrins was recognised in the form of natalizumab, for both the treatment of multiple sclerosis and CD (von Andrian 2003). However, the contemporary use of natalizumab for CD has been limited by the risk of progressive multifocal leukoencephalopathy (Bloomgren 2012). Vedolizumab specifically inhibits the α4β7 integrin from binding to MAdCAM‐1, a molecule selectively expressed in the gastrointestinal tract (Butcher 1996). This more selective mechanism of action should theoretically reduce the likelihood of progressive multifocal leukoencephalopathy.

### How the intervention might work

The value of α4β7 as a target in the treatment of IBDs was initially demonstrated in animal colitis models (Hesterberg 1996). Furthermore, a previous Cochrane Review suggested vedolizumab is effective in inducing and maintaining remission in moderate‐to‐severe ulcerative colitis (UC) (Bickston 2014).

### Why it is important to do this review

This review aims to highlight the efficacy and risks of vedolizumab in CD compared to placebo. A prior systematic review in 2014 concluded that vedolizumab was more effective than placebo as an induction and maintenance therapy for IBD, which includes CD and UC (Wang 2014). Similarly, another systematic review and meta‐analysis in 2014 concluded that vedolizumab was superior to placebo for inducing remission and response in UC (Bickston 2014).

Moćko 2016 previously published a systematic review and meta‐analysis for the effectiveness and safety of vedolizumab in Crohn's disease and identified two studies for quantitative analysis. This analysis was limited to outcomes within the induction phase of vedolizumab treatment (induction of remission, clinical response and safety).

This is an up‐to‐date review and analysis for outcomes in both the induction and maintenance phases of vedolizumab in CD.

## Objectives

To evaluate the benefits and harms of vedolizumab versus placebo for the induction and maintenance of remission in people with Crohn's disease.

## Methods

### Criteria for considering studies for this review

#### Types of studies

We included randomised controlled trials (RCTs) and quasi‐RCTs (where treatment allocations were determined by non‐randomised methods). This included published conference abstracts.

#### Types of participants

We included adults, children, or both with CD (defined by clinical, histological or endoscopic criteria) in this review. We also considered studies where only a subset of participants met the inclusion criteria.

#### Types of interventions

We included studies comparing vedolizumab to placebo at any dose, frequency and route of administration (subcutaneous and intravenous (IV)).

#### Types of outcome measures

We analysed studies which looked at induction of remission (induction studies) separately to studies where the primary outcome was to maintain remission (maintenance studies).

##### Primary outcomes

For induction studies:

- proportion of people who achieved clinical remission

For maintenance studies:

- proportion of people who maintained clinical remission

##### Secondary outcomes

For induction studies:

- rate of clinical response
- rate of adverse events (defined by study authors)
- rate of serious adverse events (defined by study authors)
- rate of surgery
- rate of endoscopic remission
- rate of endoscopic response

For maintenance studies:

- rate of adverse events (defined by study authors)
- rate of serious adverse events (defined by study authors)
- rate of surgery
- rate of endoscopic remission
- rate of endoscopic response

###### Timing of outcome measurement

For induction studies, outcomes were measured after induction vedolizumab and prior to commencement of maintenance therapy, as defined by the authors.

For maintenance studies, outcomes were measured at or after 52 weeks where available.

### Search methods for identification of studies

The search strategies are reported in Appendix 1.

#### Electronic searches

We searched the following electronic databases:

- Cochrane Central Register of Controlled Trials (CENTRAL via Cochrane Library; Issue 1, 2022);
- Ovid MEDLINE (1946 to 30 November 2022);
- Ovid Embase (1980 to 30 November 2022);
- World Health Organization International Clinical Trials Registry Platform (on 30 November 2022);
- ClinicalTrials.gov (clinicaltrials.gov; on 30 November 2022).

#### Searching other resources

We conducted further searches based on reference lists of identified studies. We searched key terms in major conference abstracts (Digestive Diseases Week, United European Gastroenterology Week, European Crohn's and Colitis Organisation Congress) between December 2008 and November 2022 to identify additional unpublished trials.

We restricted the search to 2008 onwards as the first industry sponsored phase 2 trial in CD was published in this year (Feagan 2008).

### Data collection and analysis

#### Selection of studies

Two review authors (SH and AK) independently screened titles and abstracts from our literature search for relevance based on our inclusion criteria. We retrieved and reviewed the full text of potentially relevant publications. We resolved any disagreements between review authors regarding inclusion criteria through discussion with a third review author (RB).

#### Data extraction and management

We extracted the following data onto a piloted data collection form.

- General information: title, author, year of publication, journal
- Study information: study design, setting, inclusion/exclusion criteria, type of disease activity scoring instrument used
- Population characteristics: baseline characteristics (age, sex, disease distribution, disease duration, concomitant medications, prior exposure to anti‐TNF agents), number of participants recruited, total number of participants screened and randomised to each group
- Intervention characteristics: dose and schedule of vedolizumab, use of adjunct therapies (corticosteroids, other immunomodulators), duration of treatment
- Follow‐up: length of follow‐up, withdrawals, number of participants lost to follow‐up
- Outcomes: primary and secondary (see Primary outcomes; Secondary outcomes)

#### Assessment of risk of bias in included studies

Two review authors (SH and AK) independently assessed the methodological quality of each study using the Cochrane RoB 1 tool (Higgins 2017). We judged the following factors at high, low or unclear risk of bias:

- sequence generation (i.e. was the allocation sequence adequately generated?);
- allocation sequence concealment (i.e. was allocation adequately concealed?);
- blinding (i.e. was knowledge of the allocated intervention adequately prevented during the study?);
- incomplete outcome data (i.e. were incomplete outcome data adequately addressed?);
- selective outcome reporting (i.e. were reports of the study free of suggestion of selective outcome reporting?);
- other potential sources of bias (i.e. was the study apparently free of other problems that could have put it at high risk of bias?).

#### Measures of treatment effect

We analysed data using Review Manager Web on an intention‐to‐treat basis (RevMan Web 2020). Primary and secondary outcomes were all dichotomous, and we expressed results as risk ratios (RR) with corresponding 95% confidence intervals (CI). The investigators of included studies set the definitions for clinical and endoscopic remission.

#### Unit of analysis issues

When studies reported multiple observations for the same outcome, we combined the outcomes for fixed intervals of follow‐up (e.g. clinical remission at eight weeks). We included cross‐over trials if data were available from the first phase of the study (i.e. before any cross‐over). Where studies allocated participants to more than one treatment arm, then we pooled these arms for the primary analysis. Although some studies may have reported more than one efficacy or safety event per participant, the primary analysis considered the proportion of participants who experienced at least one event.

Cluster‐randomised trials were eligible for inclusion in this review. If any cluster‐randomised trials were identified, we intended to adjust for clustering using an estimate of the intraclass correlation coefficient as described in the *Cochrane Handbook for Systematic Reviews of Interventions* (Higgins 2017).

#### Dealing with missing data

For studies with missing or unclear data, we contacted the study authors by e‐mail. We counted dichotomous data that remained missing or unclear as a treatment failure, in line with the intention‐to‐treat principle. For missing continuous data, we planned to conduct an available‐case analysis. Where appropriate, we conducted sensitivity analyses to assess the impact of including unclear data on the effect estimate.

We contacted the study authors by e‐mail to follow‐up other missing information, such as study design and standard deviations.

#### Assessment of heterogeneity

We planned to assess for heterogeneity first by visual inspection of forest plots. We observed the presence of statistical heterogeneity based on the Chi^2^ test (with a P value of 0.10 considered significant). We then aimed to quantify statistical heterogeneity using the I^2^ statistic, in line with the *Cochrane Handbook for Systematic Reviews of Interventions* (Higgins 2017). We based our interpretation of the I^2^ statistic on:

- 0% to 40%: might not be important;
- 30% to 60%: may represent moderate heterogeneity;
- 50% to 90%: may represent substantial heterogeneity;
- 75% to 100%: considerable heterogeneity.

In situations of moderate‐to‐considerable heterogeneity, we aimed to exclude visually obvious outliers if there were methodological or clinical factors present in those studies to explain the heterogeneity.

#### Assessment of reporting biases

We evaluated potential reporting bias by comparing outcomes listed in protocols to published manuscripts. If the protocols were unavailable, we compared outcomes listed in the methods section of published manuscripts to those described in the results section. If there were a sufficient number of studies included (i.e. more than 10) in the pooled analyses, we planned to investigate potential publication bias using funnel plots.

#### Data synthesis

We combined data for meta‐analysis when we determined, by consensus, that participant groups, interventions and outcomes were sufficiently similar. For binary outcomes, we calculated the pooled RR and 95% CIs. We used a random‐effects model to pool studies.

#### Subgroup analysis and investigation of heterogeneity

We performed subgroup analysis for the primary outcomes according to prior TNF inhibitor failure, compared to those who had not previously failed TNF inhibitors.

#### Sensitivity analysis

Sensitivity analyses examined the impact of the following variables on the pooled effect.

- Random‐effects versus fixed‐effect model
- Only including studies at low risk of bias across all domains (selection, performance, detection, attrition and reporting bias)
- Loss to follow‐up (greater than 10% versus less than 10%)

#### Summary of findings and assessment of the certainty of the evidence

We assessed the certainty of the total body of evidence using the GRADE criteria (Schünemann 2011). Evidence from RCTs was considered high certainty and was downgraded according to:

- study limitations (risk of bias);
- indirectness;
- inconsistency (unexplained heterogeneity);
- imprecision:
  - for the optimum information size calculation, we used a previously published resource that offers data on appropriate sample sizing for trials in this field (Gordon 2021);
  - for effects that crossed the line of no effect, we used the size of CIs to judge for imprecision. As there is no existing published resource in the field to judge imprecision based on CI sizes, we determined the following ranges following discussion within our review team:
    - for serious adverse events, we defined a narrow CI as within ± 10 per 1000 events, a moderately narrow CI as within ± 20 per 1000 events and anything greater than ± 20 per 1000 events as a wide CI;
    - for overall adverse events, we defined a narrow CI as within ± 30 per 1000 events, a moderately narrow CI as within ± 50 per 1000 events and anything greater than ± 50 per 1000 events as a wide CI;
- publication bias.

We classified the overall certainty of the evidence for each outcome as: high certainty (i.e. further research is very unlikely to change our confidence in the estimate of effect); moderate certainty (i.e. further research is likely to have an important impact on our confidence in the estimate of effect and may change the estimate); low certainty (i.e. further research is very likely to have an important impact on our confidence in the estimate of effect and is likely to change the estimate); or very low certainty (i.e. we are very uncertain about the estimate).

We used GRADEpro GDT to produce the summary of findings tables. The tables included the following key outcomes.

##### Induction studies

- Induction of clinical remission
- Induction of clinical response
- Adverse events
- Serious adverse events

##### Maintenance studies

- Maintenance of clinical remission
- Adverse events
- Serious adverse events

## Results

### Description of studies

The results of the search are presented in the PRISMA flow diagram (Figure 1). Study characteristics are included in Table 3 and Table 4.

**1:**
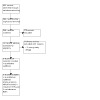


**1:** Induction studies' characteristics

**Study ID**	**Numbers randomised group**	**Trial registration number**	**Published protocol**	**Do the outcomes reported match the protocol or trial register?**
Feagan 2008	185 participants randomised into 3 arms. IV placebo (n = 58) at days 1 and 29 IV vedolizumab (MLN0002) 0.5 mg/kg (n = 62) at days 1 and 29 IV vedolizumab (MLN0002) 2 mg/kg (n = 65) at days 1 and 29	Not found	Not found	N/A
Sandborn 2013 – Induction Phase	368 participants randomised into 2 arms in a 3:2 ratio (intervention:placebo) IV placebo (n = 148) at weeks 0 and 2 IV vedolizumab 300 mg (n = 220) at weeks 0 and 2	ClinicalTrials.gov number: NCT00783692	Yes	Yes
Sands 2014	315 participants randomised into 2 arms IV placebo (n = 207) at weeks 0, 2 and 6 IV vedolizumab 300 mg (n = 209) at weeks 0, 2 and 6	ClinicalTrials.gov number: NCT01224171	Not found	N/A
Watanabe 2020 – Induction Phase	157 participants randomised into 2 arms IV placebo (n = 78) at weeks 0, 2 and 6 IV vedolizumab 300 mg (n = 79) at weeks 0, 2 and 6	ClinicalTrials.gov number: NCT02038920	Not found	N/A

IV: intravenous; n: number of participants; N/A: not applicable.

**2:** Maintenance studies' characteristics

**Study ID**	**Numbers randomised**	**Trial registration number**	**Published protocol**	**Do the outcomes reported match the protocol or trial register?**
Sandborn 2013 – Maintenance Phase	461 participants randomised into 3 arms IV placebo (n = 153) between weeks 6 and 52 IV vedolizumab 300 mg 8 weekly (n = 154) between weeks 6 and 52 IV vedolizumab 300 mg 4‐weekly (n = 154) between weeks 6 and 52	ClinicalTrials.gov number: NCT00783692	Yes	Yes
Vermeire 2021	409 participants randomised into 2 arms in a 2:1 ratio (intervention:placebo) Subcutaneous vedolizumab 108 mg (n = 275), 2‐weekly between weeks 6 and 50 Subcutaneous placebo (n = 134), 2‐weekly between weeks 6 and 50	ClinicalTrials.gov number: NCT02611817	Yes	Yes (matches ClinicalTrials.gov protocol)
Watanabe 2020 – Maintenance Phase	24 participants randomised into 2 arms IV vedolizumab 300 mg (n = 12) at week 14 then every 8 weeks until week 54 IV placebo (n = 12) at week 14 then every 8 weeks until week 54	ClinicalTrials.gov number: NCT02038920	Yes	Yes

IV: intravenous; n: number of participants.

#### Results of the search

Our electronic search identified 665 records (updated to 30 November 2022). After removing duplicates, 588 records remained for review of titles and abstracts. We excluded 575 records at this stage. We assessed the full text of the remaining 13 records, and excluded three full‐text articles, due to the wrong study design (Pipek 2020; Sandborn 2014; Vermeire 2017; Characteristics of excluded studies table).

Five studies (comprised of 10 records) met the inclusion criteria for the review (Characteristics of included studies table). For the purpose of analysis, we considered induction studies separately to maintenance studies. This resulted in four induction RCTs (Feagan 2008; Sandborn 2013 – Induction Phase; Sands 2014; Watanabe 2020 – Induction Phase), and three maintenance RCTs (Sandborn 2013 – Maintenance Phase; Vermeire 2021; Watanabe 2020 – Maintenance Phase).

There are no studies awaiting classification or ongoing.

#### Included studies

A summary of key characteristics across the included studies is shown in Table 3, Table 5, Table 6 (induction studies) and Table 4, Table 7, Table 8 (maintenance studies), and the Characteristics of included studies table.

**3:** Induction studies' intervention details

**Study ID**	**Intervention group**	**Control group**	**Previous experience of biological therapy**	**Medications up to study beginning**	**Medications that had to be discontinued prior to starting study**	**Mandatory medications per study protocol**	**Concomitant medications during study**
Feagan 2008	2 intervention groups: IV vedolizumab (MLN0002) 0.5 mg/kg at days 1 and 29 IV vedolizumab (MLN0002) 2 mg/kg at days 1 and 29	IV placebo at days 1 and 29 (type of placebo infusion not stated)	No – prior biological therapy for CD were ineligible for inclusion	Mesalamine use Placebo group: 47% IV MLN0002 0.5 mg/kg group: 55% IV MLN0002 2 mg/kg group: 42%	People requiring ciclosporin, immunosuppressives, systemic corticosteroids, heparin, non‐steroidal anti‐inflammatory drugs within 30 days before screening **were excluded**	N/A	Mesalamine use Placebo group: 47% IV MLN0002 0.5 mg/kg group: 55% IV MLN0002 2 mg/kg group: 42%
Sandborn 2013 – Induction Phase	IV vedolizumab 300 mg at weeks 0 and 2	IV placebo at weeks 0 and 2 (type of placebo infusion not stated)	Yes (but not completely stated) Prior anti‐TNF failure Placebo group 4.3% Vedolizumab group 10.5% Prior immunosuppressive failure but not prior anti‐TNF failure Placebo group 10% Vedolizumab group 17.1%	Concomitant corticosteroids Placebo group: 30.4% Vedolizumab group: 30.5% Concomitant immunosuppressive agents only Placebo group: 16.9% Vedolizumab group: 16.8% Glucocorticoids and immunosuppressive agents Placebo group: 17.6% Vedolizumab group 17.3%	Exclusion criteria Adalimumab use within 30 days Infliximab use within 60 days Certolizumab pegol use within 60 days Use of investigational or approved agents within 60 days 5‐ASA or steroid enema or suppository use within 2 weeks of the first dose	N/A	Concomitant corticosteroids Placebo group: 30.4% Vedolizumab group: 30.5% Concomitant immunosuppressive agents only Placebo group: 16.9% Vedolizumab group: 16.8% Glucocorticoids and immunosuppressive agents Placebo group: 17.6% Vedolizumab group 17.3%
Sands 2014	IV vedolizumab 300 mg at weeks 0, 2 and 6	IV placebo at weeks 0, 2 and 6 (250 mL of 0.9% sodium chloride)	Yes Participants were considered for the primary outcome if they had failed TNF antagonists. Prior TNF antagonist failure Placebo group 76% Vedolizumab group 76%	Corticosteroid use Placebo group: 52% Vedolizumab group: 53% Immunosuppressive use Placebo group: 33% Vedolizumab group: 34% Mesalamine use Placebo group: 29% Vedolizumab group: 33%	N/A	All participants had experienced inadequate response, loss of response or intolerance to TNF antagonists, immunosuppressives or corticosteroids within the past 5 years. The primary efficacy analysis was restricted to participants with prior TNF antagonist failure.	Corticosteroid use Placebo group: 52% Vedolizumab group: 53% Immunosuppressive use Placebo group: 33% Vedolizumab group: 34% Mesalamine use Placebo group: 29% Vedolizumab group: 33%
Watanabe 2020 – Induction Phase	IV vedolizumab 300 mg at weeks 0, 2 and 6	IV placebo at weeks 0, 2 and 6	Yes Prior TNF antagonist failure Placebo group 78.2% Vedolizumab group 75.9%	5‐ASA Placebo group 75.6% Vedolizumab group 81.0% Oral corticosteroids and no immunomodulators Placebo group 9.0% Vedolizumab group 16.5% Oral corticosteroids and immunomodulators Placebo group 14.1% Vedolizumab group 11.4%	Participants who started oral 5‐ASAs, probiotics, antibiotics for CD treatment, or oral corticosteroids (≤ 30 mg/day) within 13 days before the first dose of the study drug were excluded.	N/A	5‐ASA Placebo group 75.6% Vedolizumab group 81.0% Oral corticosteroids and no immunomodulators Placebo group 9.0% Vedolizumab group 16.5% Oral corticosteroids and immunomodulators Placebo group 14.1% Vedolizumab group 11.4%

5‐ASA: 5‐aminosalicylic acid; CD: Crohn's disease; IV: intravenous; N/A: not applicable; TNF: tumour necrosis factor.

**4:** Induction studies' primary and secondary outcomes

**Study ID**	**Clinical remission**	**Clinical response**	**Endoscopic remission**	**Total numbers of participants with adverse events**	**Serious adverse events**	**Surgery**
Feagan 2008	At day 57 IV vedolizumab (MLN0002) 0.5 mg/kg group: 19/62 (30%) IV vedolizumab (MLN0002) 2 mg/kg group: 24/65 (37%) Placebo group: 12/58 (21%)	At day 57 IV MLN0002 0.5 mg/kg group: 30/62 (49%) IV MLN0002 2 mg/kg group: 34/65 (53%) Placebo group: 24/58 (41%)	N/A	≥ 1 adverse event IV MLN0002 0.5 mg/kg group: 58/62 (94%) IV MLN0002 2 mg/kg group: 59/65 (91%) Placebo group: 50/58 (86%)	≥ 1 serious adverse event IV MLN0002 0.5 mg/kg group: 6/62 (10%) IV MLN0002 2 mg/kg group: 10/65 (15%) Placebo group: 10/58 (17%)	N/A
Sandborn 2013 – Induction Phase	At week 6 IV vedolizumab group: 32/220 (14.5%) IV placebo group: 10/148 (6.8%)	At week 6 IV vedolizumab group: 69/220 (31.4%) IV placebo group: 38/148 (25.7%)	N/A	IV vedolizumab group: 124/220 (56%) IV placebo group: 88/148 (59%)	IV vedolizumab group: 20/220 (9%) IV placebo group: 9/148 (6%)	N/A
Sands 2014	At week 6 IV vedolizumab group: 40/209 (19.1%) IV placebo group: 25/207 (12.1%)	At week 6 IV vedolizumab group 82/209 (39.2%) IV placebo group: 47/207 (22.7%)	N/A	IV vedolizumab group 117/209 (56%) IV placebo group 124/207 (60%)	IV vedolizumab group 13/209 (6%) IV placebo group: 16/207 (8%)	N/A
Watanabe 2020 – Induction Phase	At week 6 IV vedolizumab group: 11/79 (13.9%) IV placebo group: 10/78 (12.8%)	At week 6 IV vedolizumab group: 19/79 (24.1%) IV placebo group: 10/78 (12.8%)	N/A	IV vedolizumab group: 49/79 (62%) IV placebo group: 42/78 (53.8%)	IV vedolizumab group: 8/79 (10.1%) IV placebo group: 10/78 (12.8%)	N/A

IV: intravenous; N/A: not applicable.

**5:** Maintenance studies' intervention details

**Study ID**	**Intervention group**	**Control group**	**Previous experience of biological therapy**	**Medications up to study beginning**	**Medications that had to be discontinued prior to starting study**	**Mandatory medications per study protocol**	**Concomitant medications during study**
Sandborn 2013 – Maintenance Phase	2 intervention groups IV vedolizumab 300 mg 8 weekly IV vedolizumab 300 mg 4 weekly	IV placebo (frequency or type of infusion not stated)	Yes (but non‐TNF biologicals not stated) Prior anti‐TNF use Placebo group: 82/153 (54%) IV vedolizumab 300 mg 8 weekly group: 88/154 (57%) IV vedolizumab 300 mg 4 weekly group: 83/154 (54%)	Concomitant corticosteroids only Placebo group: 56/153 (37%) IV vedolizumab 300 mg 8‐weekly group: 59/154 (38%) IV vedolizumab 300 mg 4‐weekly group: 58/154 (38%) Concomitant immunosuppressives only Placebo group: 23/153 (15%) IV vedolizumab 300 mg 8‐weekly group: 27/154 (18%) IV vedolizumab 300 mg 4‐weekly group: 31/154 (20%) Corticosteroids and immunosuppressives: Placebo group: 26/153 (17%) IV vedolizumab 300 mg 8‐weekly group: 23/154 (15%) IV vedolizumab 300 mg 4‐weekly group: 22/154 (14%)	Exclusion criteria Adalimumab use within 30 days Infliximab use within 60 days Certolizumab pegol use within 60 days Use of investigational or approved agents within 60 days 5‐ASA or steroid enema or suppository use within 2 weeks of the first dose	N/A	Concomitant corticosteroids only Placebo group: 56/153 (37%) IV vedolizumab 300 mg 8‐weekly group: 59/154 (38%) IV vedolizumab 300 mg 4‐weekly group: 58/154 (38%) Concomitant immunosuppressives only Placebo group: 23/153 (15%) IV vedolizumab 300 mg 8‐weekly group: 27/154 (18%) IV vedolizumab 300 mg 4‐weekly group: 31/154 (20%) Corticosteroids and immunosuppressives Placebo group: 26/153 (17%) IV vedolizumab 300 mg 8‐weekly group: 23/154 (15%) IV vedolizumab 300 mg 4‐weekly group: 22/154 (14%)
Vermeire 2021	Subcutaneous vedolizumab 108 mg administered 2‐weekly between weeks 6 and 50	Subcutaneous placebo administered 2‐weekly between weeks 6 and 50	People with previous exposure to approved or investigational anti‐integrin antibodies (e.g. natalizumab, efalizumab, etrolizumab, abrilumab (AMG 181)), antimucosal addressin cell adhesion molecule‐1 antibodies or rituximab and vedolizumab were excluded Prior anti‐TNF use Placebo group: 71/134 (53%) Vedolizumab group: 168/275 (61.1%)	Prior use of immunomodulator only Placebo group: 4/134 (3%) Vedolizumab group: 16/275 (5.8%) Prior use of oral corticosteroids only Placebo group: 23/134 (17.2%) Vedolizumab group: 67/275 (24.4%) Prior use of oral corticosteroids and immunomodulators Placebo group: 103/134 (76.9%) Vedolizumab group: 189/275 (68.7%)	Excluded if topical (rectal) treatment with 5‐ASAs or corticosteroid enemas/suppositories within 2 weeks of administration of first dose of study drug. Excluded if receipt of any investigational or approved biological/biosimilar within 60 days or 5 half‐lives of screening, or receipt of any non‐permitted investigational or approved non‐biological therapies within 30 days or 5 half‐lives of screening. Oral 5‐ASA probiotics and antibiotics were permitted if doses were stable for 2 weeks before the first dose of the study and remained stable throughout the study. Azathioprine, 6‐mercaptopurine or methotrexate could be continued if the participant's dose had been stable for 8 weeks before start of study	N/A	Only immunomodulators Placebo group: 34/134 (25.4%) Vedolizumab group: 51/275 (18.5%) Only corticosteroids Placebo group: 31/134 (23.1%) Vedolizumab group: 64/275 (23.3%) Immunomodulators and corticosteroids Placebo group: 13/134 (9.7%) Vedolizumab group: 31/275 (11.3%)
Watanabe 2020 – Maintenance Phase	IV vedolizumab 300 mg at week 14 then every 8 weeks until week 54	IV placebo at week 14 then every 8 weeks until week 54	Prior anti‐TNF use Placebo group: 7/12 (58.3%) Vedolizumab group: 8/12 (66.7%)	Prior immunomodulators failure Placebo group: 6/12 (50%) Vedolizumab group: 7/12 (58.3%) Prior corticosteroids failure Placebo group: 4/12 (33.3%) Vedolizumab group: 4/12 (33.3%)	People who started oral 5‐ASAs, probiotics, antibiotics for CD treatment, or oral corticosteroids (≤ 30 mg/day) within 13 days before the first dose of the study drug were excluded.	N/A	5‐ASA Placebo group: 11/12 (91.7%) Vedolizumab group: 8/12 (66.7%) Oral corticosteroids and no immunomodulators Placebo group: 3/12 (25%) Vedolizumab group: 2/12 (16.7%) No oral corticosteroids or immunomodulators Placebo group: 3/12 (25%) Vedolizumab group: 1/12 (8.3%) Oral corticosteroids and immunomodulators Placebo group: 0/12 (0%) Vedolizumab group: 3/12 (25%)

5‐ASA: 5‐aminosalicylic acid; IV: intravenous; TNF: tumour necrosis factor.

**6:** Maintenance studies' primary and secondary outcomes

**Study ID**	**Clinical relapse**	**Serious adverse events**	**Total adverse events**	**Endoscopic relapse**	**Surgery**
Sandborn 2013 – Maintenance Phase	Clinical remission at week 52 IV vedolizumab 8‐weekly group: 60/154 (39%) IV vedolizumab 4‐weekly group: 56/154 (36.4%) IV placebo group: 33/153 (21.6%) Clinical relapse at week 52 IV vedolizumab 8‐weekly group: 94/154 (61%) IV vedolizumab 4‐weekly group: 98/154 (63.6%) IV placebo group: 120/153 (78.4%)	IV vedolizumab 8‐weekly group: 28/154(18%) IV vedolizumab 4‐weekly group: 25/154 (16%) IV placebo group: 23/153 (16%)	IV vedolizumab 8‐weekly group: 135/154(88%) IV vedolizumab 4‐weekly group: 130/154 (84%) IV placebo group: 128/153 (84%)	N/A	N/A
Vermeire 2021	Clinical remission at week 52 Vedolizumab group: 132/275 (48%) Placebo group: 46/134 (34.3%) Clinical relapse at week 52 Vedolizumab group: 143/275 (52%) Placebo group: 88/134 (65.7%)	Vedolizumab group: 23/275 (8.4%) Placebo group: 14/134 (10.4%)	Vedolizumab group: 202/275 (73.5%) Placebo group: 102/134 (76.1%)	N/A	N/A
Watanabe 2020 – Maintenance Phase	Clinical remission at week 60 Vedolizumab group: 5/12 (41.7%) Placebo group: 2/12 (16.7%) Clinical relapse at week 60 Vedolizumab group: 7/12 (58.3%) Placebo group: 10/12 (83.3%)	Vedolizumab group: 2/12 (16.7%) Placebo group: 4/12 (33.3%)	Vedolizumab group: 9 /12 (75%) Placebo group: 10/12 (83.3%)	N/A	N/A

IV: intravenous; N/A: not applicable.

##### Study design

This review included five RCTs. Several trials had separate induction and maintenance phases, where a second randomisation would occur at the maintenance phase amongst those who had responded to induction therapy. Therefore, we analysed these studies as separate induction and maintenance trials.

###### Induction studies

Four RCTs were induction studies (Feagan 2008; Sandborn 2013 – Induction Phase; Sands 2014; Watanabe 2020 – Induction Phase). All were multicentre studies; Sandborn 2013 – Induction Phase and Sands 2014 were conducted across multiple countries, while Feagan 2008 was based in Canada and Watanabe 2020 – Induction Phase was a Japanese cohort.

###### Maintenance studies

Three RCTs were maintenance trials (Sandborn 2013 – Maintenance Phase; Vermeire 2021; Watanabe 2020 – Maintenance Phase). Sandborn 2013 – Maintenance Phase and Watanabe 2020 – Maintenance Phase had an earlier randomised induction phase that then followed into a second randomisation for the maintenance phase. Vermeire 2021 only included a randomised maintenance phase with an open‐label, non‐placebo‐controlled induction phase. This study was multicentre across multiple different countries.

##### Participants

For the induction phase, the studies included 1025 participants with active CD. For the maintenance phase, the studies included 895 participants who had active CD and had then developed a clinical response to induction vedolizumab.

##### Interventions

###### Induction studies

- Feagan 2008 compared IV vedolizumab (reported as MLN0002) 0.5 mg/kg and 2 mg/kg groups versus placebo, administered at days one and 29.
- Sandborn 2013 – Induction Phase compared IV vedolizumab 300 mg versus placebo, administered at weeks zero and two.
- Sands 2014 compared IV vedolizumab 300 mg versus placebo, administered at weeks zero, two and six.
- Watanabe 2020 – Induction Phase compared IV vedolizumab 300 mg versus placebo, given at weeks zero, two and six.

###### Maintenance studies

- Sandborn 2013 – Maintenance Phase compared IV vedolizumab 300 mg in an eight‐weekly group, to a four‐weekly group and a placebo group, amongst those who had responded in an induction phase, measured at week six.
- Vermeire 2021 compared subcutaneous vedolizumab 108 mg to a placebo group, administered two weekly, amongst those who had responded to an induction phase of IV vedolizumab measured at week six.
- Watanabe 2020 – Maintenance Phase compared IV vedolizumab 300 mg at an eight‐weekly interval to placebo, amongst those who had responded to an induction phase of IV vedolizumab measured at week 10.

##### Control/comparisons

All studies (both induction and maintenance) were placebo controlled.

###### Induction studies

- Feagan 2008 and Watanabe 2020 – Induction Phase did not specify the type of IV placebo administered.
- Sands 2014 and Sandborn 2013 – Induction Phase used 250 mL of 0.9% sodium chloride for the placebo group.

###### Maintenance studies

- Sandborn 2013 – Maintenance Phase used 250 mL of 0.9% sodium chloride for the placebo group.
- Watanabe 2020 – Maintenance Phase used an unspecified IV placebo. Vermeire 2021 used an unspecified subcutaneous placebo.

##### Concurrent therapies

###### Induction studies

- Feagan 2008 allowed participants to use concurrent mesalamine.
- Sandborn 2013 – Induction Phase allowed participants to use concomitant corticosteroids and immunosuppressive agents, but not recent biological agents, mesalamine or topical glucocorticoids.
- Sands 2014 allowed participants to use corticosteroids, immunosuppressives or mesalamine.
- Watanabe 2020 – Induction Phase allowed participants to use corticosteroids, immunosuppressives or mesalamine.

###### Maintenance studies

- Sandborn 2013 – Maintenance Phase allowed participants to use concomitant corticosteroids and immunosuppressive agents, but not recent biological agents, 5‐aminosalicylic acid or topical glucocorticoids.
- Vermeire 2021 allowed participants to use concomitant corticosteroids, mesalamine or immunosuppressive agents.
- Watanabe 2020 – Maintenance Phase allowed participants to use corticosteroids, immunosuppressives or mesalamine.

##### Disease activity

All studies reported disease activity at the beginning of all induction and maintenance phases. All the induction studies required at least moderate disease activity (Crohn's Disease Activity Index (CDAI) of 220 or greater). All the maintenance studies required a clinical response to induction vedolizumab, defined by a CDAI reduction of 70 or greater.

###### Induction studies

In Feagan 2008, the baseline mean CDAI was 288 for the vedolizumab 0.5 mg/kg, 296 for the vedolizumab 2 mg/kg group and 288 for the placebo group. In Sandborn 2013 – Induction Phase, the baseline mean CDAI was 327 in the vedolizumab group and 325 in the placebo group. In Sands 2014, the baseline mean CDAI was 314 in the vedolizumab group and 301 in the placebo group. In Watanabe 2020 – Induction Phase, the mean CDAI was 304 in the vedolizumab group and 295 in the placebo group.

###### Maintenance studies

All three maintenance studies were preceded by an induction arm. Participants who developed a clinical response (CDAI reduction of 70 or greater) to induction therapy were then randomised for the maintenance study. In Sandborn 2013 – Maintenance Phase, the mean CDAI following induction therapy was not reported. In Vermeire 2021, the median CDAI at week six was 150.5 in the subcutaneous vedolizumab group and 147.5 in the placebo group. In Watanabe 2020 – Maintenance Phase, the mean CDAI at week 10 was 147.9 in the vedolizumab group and 149.7 in the placebo group.

##### Disease duration

All studies reported disease duration, which ranged between a mean of 7.5 years and 9.6 years.

###### Induction studies

In Feagan 2008, the mean disease duration was 8.8 years for the vedolizumab 0.5 mg/kg group, 8.0 years for the vedolizumab 2 mg/kg group and 9.1 years for the placebo group. Sandborn 2013 – Induction Phase reported a mean disease duration of 9.2 years for the vedolizumab group and 8.2 years for the placebo group. Sands 2014 reported a mean disease duration of 8.4 years for the vedolizumab group and 8.0 years for the placebo group. In Watanabe 2020 – Induction Phase, the mean disease duration was 9.0 years in the vedolizumab group and 9.1 years in the placebo group.

###### Maintenance studies

Sandborn 2013 – Maintenance Phase reported a mean disease duration of 8.4 years in the eight‐weekly vedolizumab group, 7.7 years in the four‐weekly vedolizumab group and 9.6 years in the placebo group. Vermeire 2021 reported a mean disease duration of 9.5 years for the vedolizumab group and 8.2 years for the placebo group. Watanabe 2020 – Maintenance Phase reported a mean disease duration of 9.0 years in the vedolizumab group and 7.5 years in the placebo group.

##### Extent of disease

All studies except Feagan 2008 reported extent of disease. Ileocolonic disease was the most common disease distribution amongst all reported induction and maintenance studies.

###### Induction studies

Feagan 2008 did not report disease extent or distribution. Sandborn 2013 – Induction Phase reported ileal‐only disease in 16.8% of the vedolizumab group and 14.2% of the placebo group; colon‐only disease in 28.2% of the vedolizumab group and 29.1% of the placebo group; and ileocolonic disease in 55% of the vedolizumab group and in 56.8% of the placebo group. Sands 2014 reported ileal‐only disease in 16% of the vedolizumab group and 14% of the control group; colon‐only disease in 23% of the vedolizumab group and 25% of the placebo group; and ileocolonic disease in 61% of both groups. Watanabe 2020 – Induction Phase reported ileal‐only disease in 16.5% of the vedolizumab group and 11.5% of the placebo group; colon‐only disease 13.9% of the vedolizumab group and 24.4% of the placebo group; and ileocolonic disease in 69.6% of the vedolizumab group and 64.1% of the placebo group.

###### Maintenance studies

Sandborn 2013 – Maintenance Phase reported ileal‐only disease in 19% of the eight‐weekly vedolizumab group, 22% of the four‐weekly vedolizumab group and 12% for the placebo group; colonic disease in 18% of the eight‐weekly vedolizumab, 31% of the four‐weekly vedolizumab group and 28% of the placebo group; and ileocolonic disease in 64% of the eight‐weekly vedolizumab group, 47% of the four‐weekly vedolizumab group and 59% of the placebo group. Vermeire 2021 reported ileal‐only disease in 24% in the vedolizumab group and 15.7% in the placebo group; colonic‐only disease in 20% of the vedolizumab group and 19.4% of the placebo group; ileocolonic disease in 44.4% of the vedolizumab group and 55.2% of the placebo group; and "other" disease locations in 11.3% of the vedolizumab group and 9.7% of the placebo group. Watanabe 2020 – Maintenance Phase reported ileal disease in 16.7% of both groups; colonic disease in 41.7% of the vedolizumab group and 8.3% of the placebo group; and ileocolonic disease in 41.7% of the vedolizumab group and 75% of the placebo group.

##### Age

All studies reported mean or median participant age. In the induction studies, the mean ranged between 32.6 and 38.6 years. In the maintenance studies, the mean age ranged between 34.9 and 38.2 years.

##### Funding and conflicts of interest

Takeda Pharmaceuticals funded all included studies (Feagan 2008; Sandborn 2013 – Induction Phase; Sandborn 2013 – Maintenance Phase; Sands 2014; Vermeire 2021; Watanabe 2020 – Induction Phase; Watanabe 2020 – Maintenance Phase). Feagan 2008 was funded by Millennium Pharmaceuticals, which is now known as Takeda Oncology and is a subsidiary of Takeda Pharmaceuticals. All studies reported authors' financial disclosures.

#### Excluded studies

We excluded three studies as they were not RCTs (Pipek 2020; Sandborn 2014; Vermeire 2017).

### Risk of bias in included studies

The risk of bias of included studies is displayed in Figure 2.

**2:**
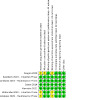


#### Induction studies

All four induction studies were randomised. Two studies had sufficient information within the study or protocol about randomisation to judge them at low risk of bias (Sandborn 2013 – Induction Phase; Sands 2014). Two studies did not mention the randomisation method and so were at unclear risk (Feagan 2008; Watanabe 2020 – Induction Phase). We wrote to the study authors and received no clarification.

Three induction studies provided sufficient information about allocation concealment to characterise them as low risk (Sandborn 2013 – Induction Phase; Sands 2014; Watanabe 2020 – Induction Phase). We contacted the authors for Feagan 2008, but received no response (unclear risk).

#### Maintenance studies

All three maintenance studies were RCTs. Sandborn 2013 – Maintenance Phase outlined the randomisation process within the protocol so was at low risk. We contacted the authors for Vermeire 2021 who confirmed the randomisation schedule was generated using interactive response technology and so was at low risk. The specific randomisation process for Watanabe 2020 – Maintenance Phase was unclear so was at unclear risk.

Allocation concealment was low risk for Sandborn 2013 – Maintenance Phase and Watanabe 2020 – Maintenance Phase where investigators were blinded to the allocations, except for unblinded pharmacists at treating sites. We contacted the authors for Vermeire 2021 who confirmed that the personnel from the vendor who had access to the randomisation schedule were not involved in the study conduct or data analysis. This was judged at low risk.

#### Blinding

All studies (induction and maintenance) were described as double‐blind.

#### Induction studies

All four induction studies were placebo controlled and at low risk of bias for blinding of participants and personnel (Feagan 2008; Sandborn 2013 – Induction Phase; Vermeire 2021; Watanabe 2020 – Induction Phase).

Sandborn 2013 – Induction Phase was at low risk of bias for blinding for outcome assessment. The authors highlighted in their protocol that the study sponsors were unblinded and analysed the data only after completion of the induction phase. We contacted the authors for Sands 2014, who confirmed that all outcome assessors were blinded to the treatment assignment and so this was at low risk. Feagan 2008 and Watanabe 2020 – Induction Phase contained insufficient information to determine risk of bias for blinding of outcome assessment. We contacted the authors but received no information (unclear risk).

#### Maintenance studies

All three maintenance studies were placebo controlled and considered low risk of bias for blinding of participants and personnel (Sandborn 2013 – Maintenance Phase; Vermeire 2021; Watanabe 2020 – Maintenance Phase). Sandborn 2013 – Maintenance Phase had two treatment arms (four‐weekly and eight‐weekly vedolizumab) and a placebo arm. To preserve the blinding, all participants were administered four‐weekly study drug or placebo.

Only Sandborn 2013 – Maintenance Phase was at low risk of blinding for outcome assessment. Procedures were in place to preserve the blinding until the completion of the maintenance phase. The other two studies contained insufficient information to determine risk of bias for blinding of outcome assessment (Vermeire 2021; Watanabe 2020 – Maintenance Phase). We contacted the authors but received no information (unclear risk).

#### Incomplete outcome data

All four induction studies and all three maintenance studies were at low risk of attrition bias. They all had low discontinuation rates, and they were balanced between groups.

#### Selective reporting

All four induction studies and all three maintenance studies were at low risk of selective reporting. All results were reported as outlined in their methods sections. Whilst all studies reported a pretrial protocol, only Sandborn 2013 – Induction Phase and Sandborn 2013 – Maintenance Phase had a published protocol that we could access. These study's results matched their registered outcomes, except one of the secondary outcomes in the Sandborn 2013 – Induction Phase protocol (CDAI‐100 response) was changed from a secondary endpoint to primary endpoint.

#### Other potential sources of bias

All studies had sufficient information on baseline characteristics between groups and were at low risk of other biases.

### Effects of interventions

See: Table 1; Table 2

See Table 1 for the results of the induction studies.

#### Induction studies

Four studies compared vedolizumab to placebo for the induction of remission in CD (Feagan 2008; Sandborn 2013 – Induction Phase; Sands 2014; Watanabe 2020 – Induction Phase). The induction phase consisted of either two or three doses of IV vedolizumab and outcomes were measured between weeks six and 10.

##### Primary outcome

###### Induction of clinical remission

In a meta‐analysis of four studies (1126 participants), 19.8% (126/635) of the vedolizumab group entered clinical remission at six to 10 weeks compared to 11.6% (57/491) of the placebo group (RR 1.61, 95% CI 1.20 to 2.17; number needed to treat for an additional beneficial outcome (NNTB) 13; Analysis 1.1).

We conducted a subgroup analysis for the primary outcome, amongst participants who had previously failed TNF inhibitor therapy and those who had not. In this analysis, the test for subgroup differences showed no evidence of a difference between the subgroups (P = 0.21). In those who had previously failed TNF inhibitor therapy (613 participants), there was no evidence of a difference in induction of clinical remission between groups (12% with vedolizumab versus 10% with placebo; RR 1.21, 95% CI 0.65 to 2.25; Analysis 1.1), although this result was affected by imprecision and some inconsistency (I^2^ = 27%). In those who had not failed anti‐TNF alpha therapy (513 participants), induction of clinical remission may be more likely in the vedolizumab compared to the placebo group (28% with vedolizumab versus 14% with placebo; RR 1.94, 95% CI 1.32 to 2.84; Analysis 1.1). However, in the absence of a difference between subgroups, we cannot be certain whether there is any true difference between subgroups.

When we used a fixed‐effect method of analysis, our conclusions remained the same. All studies were at low risk of bias and had a less than 10% loss to follow‐up, so these prespecified sensitivity analyses were not performed.

##### Secondary outcomes

###### Induction of clinical response (CDAI‐100 response)

Four induction studies recorded clinical response. Participants receiving vedolizumab were more likely to have a clinical response compared to those receiving placebo (36.9% with vedolizumab versus 24.2% with placebo; RR 1.43, CI 1.19 to 1.71; NNTB 8; 1126 participants; Analysis 1.2).

We performed subgroup analysis according to prior anti‐TNF alpha failure. There were no subgroup differences between groups (P = 0.57). In those who had previously failed TNF inhibitor therapy, participants receiving vedolizumab may be more likely to develop a clinical response compared to those receiving placebo (30.6% with vedolizumab versus 19.7% with placebo; RR 1.52, 95% CI 1.05 to 2.21; Analysis 1.2). In those who had not failed anti‐TNF alpha therapy, participants receiving vedolizumab may be more likely to develop a clinical response compared to those receiving placebo (43.4% with vedolizumab versus 30.7% with placebo; RR 1.34, 95% CI 1.05 to 1.70; Analysis 1.2).

When we used a fixed‐effect method of analysis our conclusions remained the same. We did not perform other preplanned sensitivity analyses.

###### Adverse events

All four studies recorded the proportion of participants who developed any adverse event.

For the development of one or more adverse event (treatment or non‐treatment related) during induction therapy, there was no evidence of a difference between groups (64.1% with vedolizumab versus 61.9% with placebo; RR 1.01, 95% CI 0.93 to 1.11; 1126 participants; Analysis 1.3).

When we used a fixed‐effect method of analysis our conclusions remained the same. We did not perform other preplanned sensitivity analyses.

###### Serious adverse events

All four studies recorded the proportion of participants who developed any serious adverse event.

For the development of one or more serious adverse event during induction therapy, there was no evidence of a difference between groups (9.0% with vedolizumab versus 9.2% with placebo; RR 0.91, 95% CI 0.62 to 1.33; 1126 participants; Analysis 1.4).

When we used a fixed‐effect method of analysis our conclusions remained the same. We did not perform other preplanned sensitivity analyses.

###### Surgery

No studies reported the proportion of participants requiring surgery during the induction phase.

###### Endoscopic remission

No studies reported endoscopic remission during the induction phase.

###### Endoscopic response

No studies reported endoscopic response during the induction phase.

#### Maintenance studies

See Table 2 for results of the maintenance studies.

Three studies compared vedolizumab to placebo for the maintenance of remission in CD (Sandborn 2013 – Maintenance Phase; Vermeire 2021; Watanabe 2020 – Maintenance Phase). Vermeire 2021 used subcutaneous vedolizumab whilst Sandborn 2013 – Maintenance Phase and Watanabe 2020 – Maintenance Phase used IV vedolizumab. Outcomes were recorded between weeks 52 and 60.

##### Primary outcome

###### Maintenance of clinical remission

In this pooled analysis of three studies (894 participants), 42.5% (253/595) of participants receiving vedolizumab maintained clinical remission compared to 27.1% (81/299) of participants receiving placebo at one year amongst participants with CD who had developed a clinical response to vedolizumab induction therapy (RR 1.52, 95% CI 1.24 to 1.87; NNTB 7; Analysis 2.1).

We performed subgroup analysis according to prior anti‐TNF alpha failure. There were no subgroup differences between groups (P = 0.26). In those who had previously failed TNF inhibitor therapy (462 participants), maintenance of remission may be more likely in participants receiving vedolizumab compared to those receiving placebo (36.8% with vedolizumab versus 18.8% with placebo; RR 1.81, 95% CI 1.26 to 2.58; NNTB 6; Analysis 2.1). In those who had not failed TNF inhibitor therapy (432 participants), vedolizumab may be superior to placebo in maintaining clinical remission (49.1% with vedolizumab versus 34.8% with placebo; RR 1.41, 95% CI 1.10 to 1.80; NNTB 8; Analysis 2.1).

When we used a fixed‐effect method of analysis our conclusions remained the same. All studies were at low risk of bias and had a less than 10% loss to follow‐up, so these prespecified sensitivity analyses were not performed.

##### Secondary outcomes

###### Adverse events

All maintenance studies recorded the proportion of participants who developed any adverse event.

For the development of one or more adverse event (treatment or non‐treatment related) during maintenance therapy, there was no evidence of a difference between groups (80.0% with vedolizumab versus 80.3% with placebo; RR 1.00, 95% CI 0.94 to 1.07; Analysis 2.2).

When we used a fixed‐effect method of analysis our conclusions remained the same. We did not perform other preplanned sensitivity analyses.

###### Serious adverse events

All maintenance studies recorded the proportion of participants who developed serious adverse events.

For the development of one or more serious adverse event during maintenance therapy, there was no evidence of a difference between groups (13.1% with vedolizumab versus 13.7% with placebo; RR 0.98, 95% CI 0.68 to 1.39; Analysis 2.3).

When we used a fixed‐effect method of analysis our conclusions remained the same. We did not perform other preplanned sensitivity analyses.

###### Surgery

No studies reported the proportion of participants requiring surgery during the maintenance phase.

###### Endoscopic remission

No studies reported endoscopic remission during the maintenance phase.

###### Endoscopic response

No studies reported endoscopic response during the maintenance phase.

## Discussion

### Summary of main results

Four induction RCTs enroling 1126 participants and three maintenance RCTs enroling 894 participants met the criteria for inclusion in this review.

#### Induction phase

- The evidence is very certain that vedolizumab is superior to placebo in inducing clinical remission in CD.
- The evidence is very certain that vedolizumab is superior to placebo in inducing a clinical response (CDAI‐100 response).
- There was no evidence of a difference in overall adverse events between vedolizumab and placebo during induction therapy, but the evidence was of moderate certainty due to moderately narrow CIs.
- There was no evidence of a difference in serious adverse events between vedolizumab and placebo during induction therapy, but the evidence was of low certainty due to imprecision from wide CIs.
- No induction studies reported the rate of endoscopic remission, endoscopic response or surgery.

#### Maintenance phase

- The evidence is very certain that vedolizumab is superior to placebo in maintaining clinical remission in CD.
- There was no evidence of a difference in overall adverse events between vedolizumab and placebo during maintenance therapy, but the evidence was of moderate certainty due to moderately narrow CIs.
- There was no evidence of a difference in serious adverse events between vedolizumab and placebo during maintenance therapy, but the evidence was of low certainty due to imprecision from sparse events.
- No maintenance studies reported the rate of endoscopic remission, endoscopic response or surgery.

### Overall completeness and applicability of evidence

We used a comprehensive peer‐reviewed search strategy at the protocol stage to minimise the likelihood of missing eligible reports (Hui 2020). We are unaware of any unpublished data related to the study question, although there is always the potential that randomised data within the grey literature have been missed.

The overall results were mostly relevant to the study question in our protocol. Within our protocol for induction studies, induction of endoscopic remission and the need for surgery were secondary outcomes that none of the included trials reported. For our maintenance studies, rate of endoscopic relapse and surgery were secondary outcomes that none of the included trials reported. The lack of endoscopic relapse assessment does somewhat limit the applicability of the evidence, particularly given the increasing recognition of mucosal healing as a target to achieve long‐term outcomes in the management of CD (De Cruz 2013; Shah 2016). Despite this, all identified induction and maintenance studies contributed to the primary outcome and the main purpose of the systematic review and meta‐analysis was met.

For both the induction and maintenance studies, there was variation in the route and dosing of vedolizumab. In contemporary clinical practice, induction dosing consists of IV vedolizumab 300 mg at weeks zero, two and six. For the induction studies, Feagan 2008 administered doses which would be considered subtherapeutic. Sandborn 2013 – Induction Phase assessed outcomes only following doses a week zero and two. Within the maintenance studies, Vermeire 2021 demonstrated subcutaneous vedolizumab was superior to placebo for the maintenance of remission after IV induction. As the underlying mechanism of action is identical to the IV form, we considered it appropriate to include these data in the overall meta‐analysis.

Our analysis highlights uncertainty as to whether there is a subgroup difference between those who had previously failed a TNF inhibitor and those who had not. For the induction of remission in the subgroup who had previously failed TNF inhibitor therapy, vedolizumab may not be superior to placebo, although this result was affected by imprecision and some inconsistency. In the maintenance studies, vedolizumab may still be superior to placebo, regardless of previous TNF inhibitor failure. It may be that there is a subclass of people with CD who respond well to vedolizumab induction therapy despite prior TNF inhibitor failure and proceed to develop a sustained response. However, the identification of this subset of patients is not yet defined, and raises the wider challenges of precision medicine through the use of biomarkers in CD (Boyapati 2016).

The timing of outcome measurement for the induction studies is worth highlighting given that vedolizumab is frequently viewed as a biological with a comparatively slower onset of action in contemporary practice. This is reflected in expert consensus from the STRIDE‐II initiative (Selecting Therapeutic Targets in Inflammatory Bowel Disease), which suggests a median time to clinical response of 11 weeks and time to clinical remission of 17 weeks (Turner 2021). By contrast, within the four induction studies included in this review, outcome measurement ranged between six and 10 weeks. Notably, Feagan 2008 and Sandborn 2013 – Induction Phase documented induction outcomes following two doses of vedolizumab while Sands 2014 and Watanabe 2020 – Induction Phase recorded outcomes after three doses. This may represent an additional explanation as to why vedolizumab may be effective at maintaining clinical remission within the TNF‐inhibitor failed subgroup, whereas there was less certainty of its effect at inducing remission in this cohort. Furthermore, it was found that vedolizumab may be effective at inducing a clinical response within this TNF‐inhibitor failed subgroup, again highlighting the potential that the assessment of clinical remission may have been conducted prematurely. However, STRIDE‐II stresses that the recommendations for onset of action are guided by a rough estimate of experts' opinion due to a paucity of high‐quality scientific evidence.

### Quality of the evidence

The overall body of evidence allows a robust conclusion regarding the objective of this review. We included four induction studies (1126 participants) and three maintenance studies (894 participants). The methodological basis for these conclusions is sound. All trials were randomised, placebo controlled and described as double‐blinded.

The RoB 1 tool suggested a low risk of bias across most domains for induction and maintenance studies. There were several areas of unknown risk of bias despite contacting the study authors for clarification. Nonetheless, these overall limitations were not serious and the evidence was not downgraded for risk of bias for any of the outcomes within the summary of findings tables.

We did not downgrade for inconsistency for any outcomes across both induction and maintenance studies. The I^2^ value was low (0% to 15%) across all outcomes. We did not downgrade for indirectness for any outcomes for either induction or maintenance studies. Specifically, there was no major indirectness with regards to the population, intervention and outcome measurement.

We downgraded twice for serious adverse events for both induction and maintenance studies due to wide CIs. We downgraded once for adverse events for both induction and maintenance studies due to moderately wide CIs.

We did not downgrade for publication bias for any outcomes. All included studies were RCTs and there were no observational data included in this review. There was an insufficient number of studies to construct a funnel plot.

### Potential biases in the review process

One area of potential bias was the changes introduced between the protocol and review stage. The major change was timing of outcome measurement. In the protocol, outcomes were to be measured at weeks six, 12 and 52 where available (Hui 2020). On subsequent review of the available trials, these were broadly divided into induction and maintenance studies with separate randomisation phases. For both induction and maintenance studies, there was some variation in timepoints for outcome measurement and so we removed the timepoints for induction study outcomes. Despite this, two of four studies representing the majority of participants were measured at week six, whilst Feagan 2008 reported results at week nine (day 57) and Watanabe 2020 – Induction Phase recorded outcomes at week 10. For maintenance studies, in the review, we determined that outcomes were most appropriate to be measured at or after 52 weeks rather than strictly at week 52. In our results for maintenance studies, two of three studies reported week 52 data while Watanabe 2020 – Maintenance Phase reported results at week 60. Overall, the variation in the timing of outcome measurement across both induction and maintenance studies was small and unlikely to impact the overall results significantly.

Another limitation of the review was the heterogeneity in intervention dosage and route (subcutaneous versus IV) between trials. Amongst the induction studies, Feagan 2008 and Sandborn 2013 – Induction Phase used vedolizumab dosing that would be considered subtherapeutic in contemporary practice. In the maintenance studies, Vermeire 2021 investigated subcutaneous vedolizumab.

Finally, we identified only one published protocol for the studies in our systematic review, and there were no major discrepancies between planned and reported outcomes. For the studies which did not have a published protocol, this introduced a theoretical risk of publication bias. However, within these trials, the interventions and outcomes described in the methods section were consistent with the results. Furthermore, the primary and secondary outcomes were consistent between trials, including the definition of induction and maintenance of remission, clinical response and adverse events.

### Agreements and disagreements with other studies or reviews

This is the first Cochrane Review to investigate the efficacy and safety of vedolizumab in CD. Our findings for induction of remission were similar to a previously published systematic review (Moćko 2016), including the uncertainty of vedolizumab compared to placebo in TNF inhibitor‐experienced participants for induction of clinical remission. The overall safety and efficacy of vedolizumab in inducing and maintaining remission in IBD (both CD and UC) was also investigated in a prior systematic review (Wang 2014). This review demonstrated vedolizumab was superior to placebo for the induction and maintenance of remission of IBD, including within a subgroup analysis of induction of remission of CD.

US guidelines support the use of vedolizumab in CD and particularly recommend its use for maintenance therapy where vedolizumab has successfully induced remission (Lichtenstein 2018). UK guidelines also support the use of vedolizumab in active CD, and specifically include patients where TNF inhibitors have previously failed (Lamb 2019). The basis of this latter recommendation is the Swedish Inflammatory Bowel Disease Registry (SWIBREG) (Eriksson 2017), which reported clinical remission of 54% at a median follow‐up of 17 months amongst a cohort of people with active CD (86% of whom had previously failed TNF inhibitor therapy). These data were notably not placebo controlled or blinded. Even at six to 10 weeks within our meta‐analysis, it should be noted that 10% of participants receiving placebo who had prior TNF inhibitor failure were in clinical remission. The available results within this meta‐analysis highlight the uncertainty of this recommendation.

The GEMINI long‐term safety study (Vermeire 2017) was an open‐label extension study to GEMINI 2 which offered four‐weekly vedolizumab as maintenance therapy. This study continued to report long‐term clinical remission rates that were statistically similar between TNF inhibitor‐failed and TNF inhibitor‐naive people at week 152. This is largely consistent with the results of our review, where vedolizumab was probably superior to placebo in maintaining clinical remission in the subgroup who had previously failed TNF inhibitors.

## Authors' conclusions

**Implications for practice**

There is high‐certainty evidence that vedolizumab is effective at inducing and maintaining clinical remission in Crohn's disease. There is low‐ to moderate‐certainty evidence that there may be no increased risk of adverse events compared to placebo.

The certainty of the evidence is primarily impacted by imprecision, due to wide confidence intervals in the estimate of the effect size.

**Implications for research**

This review highlights there is minimal need to consider further induction and maintenance studies to demonstrate the efficacy and safety of vedolizumab in Crohn's disease when people are mixed populations of those who have had prior anti‐tumour necrosis factor (TNF) exposure and those who have not.

The findings have shown that future research should investigate the role of vedolizumab in people as separate trials considering whether they had experienced prior failure with TNF‐inhibitor therapy given the observed suggestion of difference in these groups.

Furthermore, future research must also consider the efficacy and safety of vedolizumab compared in head‐to‐head trials with other biological therapies in Crohn's disease. Presently, the selection of biologicals in Crohn's disease is often based on clinical judgement as there remain very few head‐to‐head randomised controlled trials (RCT) to inform clinical decisions. To our knowledge, the unpublished SEAVUE study (ustekinumab versus adalimumab) is the only RCT to date that has reported head‐to‐head results in Crohn's disease (Irving 2021).

Clear reporting of concurrent and prior therapies from other classes is also key, such as purine analogues and corticosteroids, as this informs wider future comparisons with other trials.

Finally, endoscopic remission was a secondary endpoint that did not reveal any results in our systematic review. Mucosal healing is gaining increased acceptance as an outcome of interest in the treatment of inflammatory bowel disease and future studies should consider this as an endpoint.

Key policymakers and stakeholders need to be involved in future studies to address the evidence gaps. This is especially important with biological medications in Crohn's disease given their significant cost to individuals and healthcare systems.

## History

Protocol first published: Issue 5, 2020

## Appendices

### Appendix 1. Electronic search strategy

**MEDLINE search strategy (via Ovid)**

1. random$.tw.

2. placebo$.tw.

3. single blind.mp.

4. double blind.mp.

5. triple blind.mp.

6. Single‐Blind Method/

7. Double‐Blind Method/

8. Randomized Controlled Trial/

9. or/1‐8

10. Exp Crohn Disease

11. Crohn*.mp.

12. 10 or 11

13. Vedolizumab.mp.

14. anti‐alpha4*.mp.

15. anti alpha 4*.mp.

16. anti‐alpha4beta7*.mp.

17. alpha4beta7 antibod*.mp.

18. alpha4beta7 inhibit*.mp.

19. MLN‐02.mp.

20. MLN 02.mp.

21. MLN‐0002.mp.

22. MLN 0002.mp.

23. MLN0002.mp.

24. Entyvio.mp.

25. or/13‐24

26. 9 AND 12 AND 25

**Embase search strategy (via Ovid)**

1. random$.tw.

2. placebo$.tw.

3. single blind.mp.

4. double blind.mp.

5. triple blind.mp.

6. Single‐Blind Method/

7. Double‐Blind Method/

8. Randomized Controlled Trial/

9. or/1‐8

10. Exp Crohn Disease

11. Crohn*.mp.

12. 10 or 11

13. Vedolizumab.mp.

14. anti‐alpha4*.mp.

15. anti alpha 4*.mp.

16. anti‐alpha4beta7*.mp.

17. alpha4beta7 antibod*.mp.

18. alpha4beta7 inhibit*.mp.

19. MLN‐02.mp.

20. MLN 02.mp.

21. MLN‐0002.mp.

22. MLN 0002.mp.

23. MLN0002.mp.

24. Entyvio.mp.

25. or/13‐24

26. 9 AND 12 AND 25

**Cochrane Library (Cochrane Central Register of Controlled Trials)**

1. MeSH descriptor: [Crohn Disease]

2. Crohn*

3. Vedolizumab

4. anti‐alpha4*

5. anti alpha 4*

6. anti‐alpha4beta7*

7. alpha4beta 7 antibod*

8. alpha4beta7 inhibit*

9. MLN‐2

10. MLN 02

11. MLN‐0002

12. MLN 0002

13. MLN0002

14. Entyvio

15. #1 or #2

16. #3 or #4 or #5 or #6 or #7 or #8 or #9 or #10 or #11 or #12 or #13 or #14

17. #15 AND #16 (Limits to 'Trials')

**ClinicalTrials.gov**

Advanced search

Condition or disease: Crohn Disease

Intervention/ treatment: Vedolizumab

Study type: Interventional Studies (Clinical Trials)

Study results: All studies

**WHO ICTRP search strategy**

Advanced search

Condition: Crohn's disease OR Crohn disease

Intervention: Vedolizumab OR Entyvio OR MLN0002 OR alpha4beta7

Study results: All studies

## Data and analyses

**Comparison 1:** Induction studies – vedolizumab versus placebo

Outcome or subgroup title	No. of studies	No. of participants	Statistical method	Effect size
1.1 Induction of clinical remission	4	1126	Risk Ratio (M‐H, Random, 95% CI)	1.61 [1.20, 2.17]
1.1.1 Prior TNF inhibitor failure	3	613	Risk Ratio (M‐H, Random, 95% CI)	1.21 [0.65, 2.25]
1.1.2 No prior TNF inhibitor	4	513	Risk Ratio (M‐H, Random, 95% CI)	1.94 [1.32, 2.84]
1.2 Clinical response	4	1126	Risk Ratio (M‐H, Random, 95% CI)	1.43 [1.19, 1.71]
1.2.1 Prior TNF inhibitor failure	3	613	Risk Ratio (M‐H, Random, 95% CI)	1.52 [1.05, 2.21]
1.2.2 No TNF inhibitor failure	4	513	Risk Ratio (M‐H, Random, 95% CI)	1.34 [1.05, 1.70]
1.3 Development of ≥ 1 adverse event during induction therapy	4	1126	Risk Ratio (M‐H, Random, 95% CI)	1.01 [0.93, 1.11]
1.4 Development of ≥ 1 serious adverse event during induction therapy	4	1126	Risk Ratio (M‐H, Random, 95% CI)	0.91 [0.62, 1.33]

**Comparison 2:** Maintenance studies – vedolizumab versus placebo

Outcome or subgroup title	No. of studies	No. of participants	Statistical method	Effect size
2.1 Maintenance of clinical remission	3	894	Risk Ratio (M‐H, Random, 95% CI)	1.52 [1.24, 1.87]
2.1.1 Prior TNF inhibitor failure	3	462	Risk Ratio (M‐H, Random, 95% CI)	1.81 [1.26, 2.58]
2.1.2 No prior TNF inhibitor failure	3	432	Risk Ratio (M‐H, Random, 95% CI)	1.41 [1.10, 1.80]
2.2 Development of ≥ 1 adverse event during maintenance therapy	3	894	Risk Ratio (M‐H, Random, 95% CI)	1.00 [0.94, 1.07]
2.3 Development of ≥ 1 serious adverse event during maintenance therapy	3	894	Risk Ratio (M‐H, Random, 95% CI)	0.98 [0.68, 1.39]

## Characteristics of studies

### Characteristics of included studies [ordered by study ID]

**Table:** Feagan 2008

***Study characteristics***		
Methods	**Study design:** 3‐arm double‐blind randomised trial (designed with 2 intervention and 1 placebo arms. We grouped the intervention arms for this analysis) **Number of centres:** multicentre (21 sites) **Countries:** Canada **Study dates:** February 2000 to June 2002 **Setting:** NR	
Participants	**Inclusion criteria** Adults with endoscopic, histopathological or radiological documentation of CD of the ileum or colon (or both), and a CDAI score 220–400 at screening. Participants could receive concomitant treatment for CD with mesalamine or antibiotics provided they had been maintained on a stable dose for 2 weeks immediately before screening. **Exclusion criteria** With an ostomy, an active fistula, or evidence of fixed obstruction Requiring ciclosporin or immunosuppressants within 3 months or investigational drugs within 30 days before screening Requiring systemic corticosteroids, heparin, non‐steroidal anti‐inflammatory drugs, tube feeding, defined formula diets or parenteral alimentation Previously treated with biological therapy for CD Markedly abnormal laboratory tests (haemoglobin < 10 g/dL; white blood cell count < 3 × 10^9^/L; platelet count < 100 × 10^9^/L; serum aspartate aminotransferase, alanine aminotransferase or alkaline phosphatase > 2.5 × ULN; serum creatinine > 1.5 × ULN; positive stool for enteric pathogens or proteinuria) Unable to comply with the protocol **Baseline disease characteristics** CG = placebo IG1 = cohort 1 vedolizumab (MLN0002) 0.5 mg/kg IG2 = cohort 2 vedolizumab (MLN0002) 2.0 mg/kg Mean age at beginning of study (years): CG 34.5 (SD 11.26), IG1 36 (SD 12.67), IG2 38.5 (SD 13.07) Males: CG 51.7%, IG1 40.3%, IG2 47.7% Smokers: CG 32.8%, IG1 46.8%, IG2 41.5% Mean duration of disease since diagnosis (months): CG 109 (SD 99.3), IG1 105 (SD 99.2), IG2 96 (SD 94.8) Mean CDAI score: CG 288 (SD 45.83), IG1 288.1 (SD 48.63), IG2 296.6 (SD 55.37) Mean IBDQ score: CG 122 (SD 28), IG1 131 (SD 26), IG2 131 (SD 26) Mean CRP level (mg/L): CG 2.5 (SD 2.9), IG1 1.96 (SD 2.1), IG2 2.46 (SD 3.6) Mean time on oral or IV steroids (months): CG 3.8 (SD 6.72), IG1 2.6 (SD 5.59), IG2 3.3 (SD 6.08) Number of participants randomised per group: CG 58, IG1 62, IG2 65 Number of participants reaching end of study: CG 58, IG1 61, IG2 64	
Interventions	**CG:** IV placebo weeks 1 and 29 (agent: NR) **IG1:** vedolizumab (MLN0002) 0.5 mg/kg weeks 1 and 29 **IG2:** vedolizumab (MLN0002) 2 mg/kg weeks 1 and 29 **Duration of study:** 180 days **Measurement timepoints during study** CDAI and IBDQ were determined at each study visit (days 1, 8, 15, 29, 43, 57, 85, 113, 141, 180)	
Outcomes	**Primary outcome** Rate of clinical response at day 57 (CDAI reduction ≥ 70) **Secondary outcomes** Rate of clinical remission (CDAI ≤ 150) at day 57 Rates of clinical response and clinical remission at each study visit Rates of enhanced clinical response (CDAI reduction ≥ 100) Time to clinical response and clinical remission Change in mean CDAI and IBDQ scores Change in CRP Rate of treatment failure (defined as worsening of clinical status by investigator judgement and either a CDAI increase of ≥ 100 points up to day 29 or the need for additional drug therapy to treat worsening disease activity)	
Notes		
***Risk of bias***		
**Bias**	**Authors' judgement**	**Support for judgement**
Random sequence generation (selection bias)	Unclear risk	Process of random sequence generation not described. Contacted study authors by e‐mail but received no response.
Allocation concealment (selection bias)	Unclear risk	Study described as 'double‐blind' but details on allocation concealment not described. Contacted study authors by e‐mail (Dr Brian Feagan) but received no response.
Blinding of participants and personnel (performance bias) All outcomes	Low risk	Study was 'double‐blind' and placebo‐controlled.
Blinding of outcome assessment (detection bias) All outcomes	Unclear risk	Study was 'double‐blind' but details of blinding of outcome assessment were not described. Contacted study authors by e‐mail but received no response.
Incomplete outcome data (attrition bias) All outcomes	Low risk	Low, balanced and explained attrition.
Selective reporting (reporting bias)	Low risk	Outcomes reported as per the methods section.
Other bias	Low risk	Balanced baseline characteristics. No other concerns.

**Table:** Sandborn 2013 – Induction Phase

***Study characteristics***		
Methods	**Study design:** 2‐arm double‐blind randomised study **Number of centres:** multicentre **Countries:** multiple **Study dates:** December 2008 to May 2012 **Setting:** NR	
Participants	**Induction or maintenance study:** induction then maintenance. Maintenance trial was excluded due to it being open label to meet sample size requirements. **Active or inactive disease at beginning of study:** active (there were 2 cohorts but cohort 2 was open label, therefore it was excluded, cohort 1 was double‐blind randomised) **Inclusion criteria** Aged 18–80 years Able to voluntarily give informed consent Contraception criteria CD duration ≥ 3 months (histopathology report) or ≥ 6 months (if report unavailable) CDAI score within range and 1 of the following: CRP > 2.87 mg/L; ≥ 3 non‐anastomotic ulcerations or fecal calprotectin > 250 μg/g Ileal or colonic disease, or both Colonoscopy within 12 months for long‐standing disease Colon cancer screening up‐to‐date Inadequate response, lost response, or intolerance to corticosteroids, immunosuppressives, or TNF antagonists Stable dose of 5‐ASA Stable doses of steroids Stable doses of probiotics Antidiarrhoeal use Stable doses of azathioprine or 6‐mercaptopurine Stable dose of methotrexate Stable dose of antibiotics **Exclusion criteria** Gastrointestinal exclusion criteria Abdominal abscess or toxic megacolon Extensive colonic resection, subtotal or total colectomy > 3 small‐bowel resections or short bowel syndrome Tube feeding/formula diet/hyperalimentation within 21 days Ileostomy, colostomy or symptomatic Use of non‐biological therapies (e.g. ciclosporin, thalidomide) within 30 days Investigational use of non‐biological therapy within 30 days Investigational use of approved non‐biological therapy within 30 days Adalimumab use within 30 days Infliximab use within 60 day Certolizumab pegol use within 60 days Investigational or approved agents use within 60 days Exposure to natalizumab, efalizumab or rituximab 5‐ASA or steroid enema or suppository use within 2 weeks of first dose Clostridium infection within 60 days or other intestinal pathogen within 30 days Need for surgical intervention for CD during study Presence of adenomatous colonic polyps Colonic mucosal dysplasia Diagnosis of ulcerative colitis or indeterminate colitis Infectious disease exclusion criteria Chronic HBV or chronic HCV History of tuberculosis Missing baseline tuberculosis test results Tuberculosis on chest x‐ray within 3 months Congenital or acquired immunodeficiency Live vaccines within 30 days Extraintestinal infection within 30 days General exclusion criteria Previous exposure to vedolizumab Positive pregnancy test Any unstable major medical disorder Surgical procedure requiring general anaesthesia within 30 days History of malignancy History of major neurological disorder Positive PML subjective checklist Haemoglobin < 8 g/dL White blood cell count < 3 × 10^9^/L Lymphocyte count < 0.5 × 10^9^/L Platelet count < 100 × 10^9^/L or > 1200 × 10^9^/L Alanine aminotransferase or aspartate aminotransferase level > 3 × ULN Alkaline phosphatase level > 3 × ULN Serum creatinine level > 2 × ULN Substance abuse Active psychiatric problems Inability to attend all study visits or comply with study **Baseline disease characteristics** CG = placebo IG = cohort 1 vedolizumab 300 mg Mean age at beginning of study (years): CG 38.6 (SD 13.2), IG 36.3 (SD 11.6) Males: CG 46.6%, IG 47.7% White race: CG 83.8%, IG 82.7% Mean weight (kg): CG 68.7 (SD 18.9), IG 67.1 (SD 19.1) Current smoker: CG 23%, IG 24.5% Mean duration of disease (years): CG 8.2 (SD 7.8), IG 9.2 (SD 8.2) Mean CDAI score: CG 325 (SD 78), IG 327 (SD 71) Median CRP (mg/L): CG 13.7, IG 15.3 Median fecal calprotectin (μg/g): CG 653, IG 852 Disease site – ileum only: CG 14.2%, IG 16.8% Disease site – colon only: CG 29.1%, IG 28.2% Disease site – ileum and colon: CG 56.8%, IG 55% Concomitant medications for CD – glucocorticoids only: CG 30.4%, IG 30.5% Concomitant medications for CD – immunosuppressive agents only: CG 16.9%, IG 16.8% Concomitant medications for CD – glucocorticoids and immunosuppressive agents: CG 17.6%, IG 17.3% Concomitant medications for CD – no glucocorticoids or immunosuppressive agents: CG 35.1%, IG 35.5% Median prednisolone equivalent dose (mg): CG 20, IG 20 Prior receipt of ≥ 1 TNF antagonist: CG 48.6%, IG 50.5% Prior failure of ≥ 1 TNF antagonist: CG 47.43%, IG 47.7% Prior failure of ≥ 2 TNF antagonist: CG 28.4%, IG 25.5% Mean haemoglobin (g/L): CG 124.7 (SD 18.6), IG 121.6 (SD 18.4) Mean white cell count (× 10^9^/L): CG 8.8 (SD 3), IG 9 (SD 3.3) Prior surgery for CD: CG 36.5%, IG 44.5% History of fistulising disease: CG 37.8%, IG 40.9% Draining fistulae at baseline: CG 15.5%, IG 17.3%	
Interventions	Interventions (induction trial) **CG:** IV placebo weeks 0 and 2 **IG:** IV vedolizumab 300 mg weeks 0 and 2 **Duration of study:** 6 weeks **Measurement timepoints during study** Study visits scheduled at weeks 0, 2, 4 and 6 Adverse events, CDAI, neurological symptoms of PML as means of questionnaires, use of concomitant medications, presence/absence of fistulae were evaluated at these visited Blood testing performed at baseline and "throughout the study"	
Outcomes	**Primary outcomes** Clinical remission defined as CDAI score ≤ 150 at week 6 CDAI‐100 response at week 6 (CDAI drop of ≥ 100) **Secondary outcomes** Mean change in CRP from baseline to week 6	
Notes	**Funding source:** Takeda Pharmaceuticals	
***Risk of bias***		
**Bias**	**Authors' judgement**	**Support for judgement**
Random sequence generation (selection bias)	Low risk	Quote: "Randomization was computer‐generated and was performed at a central location."
Allocation concealment (selection bias)	Low risk	Quote from protocol, "treatment assignments will be obtained through the interactive voice response system (IVRS) and for dose preparation according to the procedures outlined in the Study Manual. Information regarding the treatment assignments will be kept securely at Millennium per its standard operating procedures." Quote: "Randomization schedules will be generated by the Millennium Biostatistics Group and archived within the Biostatistics and Medical Writing Department of Millennium. Each patient who is qualified for treatment will be assigned a unique randomization number. The IVRS will provide treatment assignments based on these randomization numbers."
Blinding of participants and personnel (performance bias) All outcomes	Low risk	Trial was double blinded and placebo controlled. The protocol stated that all study site personnel except the investigational pharmacist or designee were blinded to the treatment assignments for the duration of the study.
Blinding of outcome assessment (detection bias) All outcomes	Low risk	Quote from protocol, "after the Induction Phase has been completed, select and pre‐specified personnel at Millennium will become unblinded to patient‐level data in order to conduct the analyses and reporting of the Induction Phase data. As these activities will occur while the Maintenance Phase is ongoing, proper procedures will be in place to protect the blind until completion of the Maintenance Phase."
Incomplete outcome data (attrition bias) All outcomes	Low risk	According to the flowchart of Supplementary Figure 1 (S1), attrition was balanced in all groups with adequate reasons provided for loss in numbers.
Selective reporting (reporting bias)	Low risk	Outcomes remained the same between published protocol and final trial. The only outcome change highlighted was CDAI‐100 response changed from being a secondary to primary endpoint.
Other bias	Low risk	Baseline characteristics reported and balanced for participants in all groups. No other apparent sources of bias.

**Table:** Sandborn 2013 – Maintenance Phase

***Study characteristics***		
Methods	**Study design:** 3‐arm double‐blind randomised trial **Number of centres:** multicentre **Countries:** multiple **Study dates:** December 2008 to May 2012 **Setting:** NR	
Participants	**Induction or maintenance study:** maintenance trial **Active or inactive disease at beginning of study:** inactive disease People who had a clinical response with vedolizumab in the 6‐week induction phase were included (CDAI ≥ 70 decrease). Combination of people randomised to vedolizumab in the induction trial (220 participants), in addition to a cohort of 747 open‐label participants **Inclusion criteria** Aged 18–80 years Able to voluntarily give informed consent Contraception criteria CD duration ≥ 3 months (histopathology report) or ≥ 6 months (if report unavailable) CDAI score within range and 1 of the following: CRP > 2.87 mg/L; ≥ 3 non‐anastomotic ulcerations or fecal calprotectin > 250 μg/g Ileal or colonic disease, or both Colonoscopy within 12 months for long‐standing disease Colon cancer screening up‐to‐date Inadequate response, lost response, or intolerance to corticosteroids, immunosuppressives, or TNF antagonists Stable dose of 5‐ASA Stable doses of steroids Stable doses of probiotics Antidiarrhoeal use Stable doses of azathioprine or 6‐mercaptopurine Stable dose of methotrexate Stable dose of antibiotics **Exclusion criteria** Gastrointestinal exclusion criteria Abdominal abscess or toxic megacolon Extensive colonic resection, subtotal or total colectomy > 3 small‐bowel resections or short bowel syndrome Tube feeding/formula diet/hyperalimentation within 21 days Ileostomy, colostomy or symptomatic Use of non‐biological therapies (e.g. ciclosporin, thalidomide) within 30 days Investigational use of non‐biological therapy within 30 days Investigational use of approved non‐biological therapy within 30 days Adalimumab use within 30 days Infliximab use within 60 day Certolizumab pegol use within 60 days Investigational or approved agents use within 60 days Exposure to natalizumab, efalizumab or rituximab 5‐ASA or steroid enema or suppository use within 2 weeks of first dose Clostridium infection within 60 days or other intestinal pathogen within 30 days Need for surgical intervention for CD during study Presence of adenomatous colonic polyps Colonic mucosal dysplasia Diagnosis of ulcerative colitis or indeterminate colitis Infectious disease exclusion criteria Chronic HBV or chronic HCV History of tuberculosis Missing baseline tuberculosis test results Tuberculosis on chest x‐ray within 3 months Congenital or acquired immunodeficiency Live vaccines within 30 days Extraintestinal infection within 30 days General exclusion criteria Previous exposure to vedolizumab Positive pregnancy test Any unstable major medical disorder Surgical procedure requiring general anaesthesia within 30 days History of malignancy History of major neurological disorder Positive PML subjective checklist Haemoglobin < 8 g/dL White blood cell count < 3 × 10^9^/L Lymphocyte count < 0.5 × 10^9^/L Platelet count < 100 × 10^9^/L or > 1200 × 10^9^/L Alanine aminotransferase or aspartate aminotransferase level > 3 × ULN Alkaline phosphatase level > 3 × ULN Serum creatinine level > 2 × ULN Substance abuse Active psychiatric problems Inability to attend all study visits or comply with study **Baseline disease characteristics** CG = placebo IG1 = IV vedolizumab 300 mg 8 weekly IG2 = IV vedolizumab 300 mg 4 weekly Mean age (years): CG 37.3 (SD 12), IG1 35.1 (SD 12.2), IG2 34.9 (SD 12.2) Males: CG 47%, IG1 44%, IG2 53% White race: CG 92%, IG1 88%, IG2 87% Weight (kg): CG 69.0 (SD 18.2), IG1 68.5 (SD 18.6), IG2 71.5 (SD 18.4) Current smoker: CG 31%, IG1 31%, IG2 25% Mean duration of disease (years): CG 9.6 (SD 8.9), IG1 8.4 (SD 7.3), IG2 7.7 (SD 6.8) Mean CDAI score: CG 325 (SD 66), IG1 326 (SD 69), IG2 317 (SD 66) Median CRP (μg/L): CG 9.8, IG1 8.6, IG2 9.8 Median fecal calprotectin (μg/g): CG 684, IG1 584, IG2 776 Disease localisation – ileal: CG 12%, IG1 19%, IG2 22% Disease localisation – colonic: CG 28%, IG1 18%, IG2 31% Disease localisation – ileocolonic: CG 59%, IG1 64%, IG2 47% Concomitant Crohn's medications – corticosteroids only: CG 37%, IG1 38%, IG2 38% Concomitant Crohn's medications – immunosuppressives only: CG 15%, IG2 18%, IG2 20% Concomitant Crohn's medications – corticosteroids and immunosuppressives: CG 17%, IG1 15%, IG2 14% Concomitant Crohn's medications – no corticosteroids or immunosuppressives: CG 31%, IG1 29%, IG2 28% Median prednisolone equivalent dose (mg): CG 20, IG1 20, IG2 20 Prior anti‐TNF use: CG 54%, IG1 57%, IG2 54% Prior failure of ≥1 TNF antagonist: CG 51%, IG1 55%, IG2 50% Prior failure of ≥2 TNF antagonist: CG 35%, IG1 30%, IG2 32% Mean haemoglobin (g/L): CG 126.5 (SD 15.1), IG1 125.1 (SD 17.4), IG2 126.7 (SD 17.3) Mean white cell count (× 10^9^/L): CG 9.1 (SD 3.4), IG1 9.2 (SD 3.4), IG2 9 (SD 3.3) Prior surgery for CD: CG 37%, IG1 37%, IG2 40% History of fistulising disease: CG 37%, IG1 31%, IG2 32% Draining fistulae at baseline: CG 12%, IG1 11%, IG2 14%	
Interventions	Interventions (maintenance trial) **CG:** IV placebo **IG1:** IV vedolizumab 300 mg 8 weekly **IG2:** IV vedolizumab 300 mg 4 weekly **Duration of study:** 46 weeks **Measurement timepoints during study:** study visits were conducted every 4 weeks during the maintenance trial. The primary and secondary outcomes were assessed at week 52 from time of induction (46 weeks into maintenance study) **Follow‐up measurements after study end:** those who had no unacceptable adverse events or did not require CD‐related surgery were continued in the open‐label GEMINI long‐term safety trial.	
Outcomes	**Primary outcomes as defined by study authors** Clinical remission at week 52 (in maintenance therapy trial) **Secondary outcomes as defined by study authors** CDAI‐100 response Glucocorticoid‐free remission (defined as clinical remission at week 52 without glucocorticoid therapy) Durable clinical remission (defined as clinical remission at ≥ 80% of study visits, including the final visit) at week 52	
Notes	**Funding source:** Millennium Pharmaceuticals **Conflicts of interest:** NR	
***Risk of bias***		
**Bias**	**Authors' judgement**	**Support for judgement**
Random sequence generation (selection bias)	Low risk	Quote: "Randomization was computer‐generated and was performed at a central location."
Allocation concealment (selection bias)	Low risk	Quote from protocol "treatment assignments will be obtained through the interactive voice response system (IVRS) and for dose preparation according to the procedures outlined in the Study Manual. Information regarding the treatment assignments will be kept securely at Millennium per its standard operating procedures." Quote: "Randomization schedules will be generated by the Millennium Biostatistics Group and archived within the Biostatistics and Medical Writing Department of Millennium. Each patient who is qualified for treatment will be assigned a unique randomization number. The IVRS will provide treatment assignments based on these randomization numbers."
Blinding of participants and personnel (performance bias) All outcomes	Low risk	Quote: "all patients and all study personnel except for those directly involved with study drug preparation will be blinded to study drug assignment for the entire study." Comment: use of placebo was described in protocol. During the maintenance phase all arms of the study (IV vedolizumab 8 weekly, 4 weekly and placebo) would receive either placebo or study drug **4 weekly** to maintain the blind. IV cover bags were also used to maintain blinding.
Blinding of outcome assessment (detection bias) All outcomes	Low risk	Quote: "after the Induction Phase has been completed, select and pre‐specified personnel at Millennium will become unblinded to patient‐level data in order to conduct the analyses and reporting of the Induction Phase data. As these activities will occur while the Maintenance Phase is ongoing, proper procedures will be in place to protect the blind until completion of the Maintenance Phase."
Incomplete outcome data (attrition bias) All outcomes	Low risk	According to the flowchart of Supplementary Figure 1 (S1), attrition was balanced in all groups with adequate reasons provided for loss in numbers.
Selective reporting (reporting bias)	Low risk	No published protocol found. According to trial registration, authors reported relevant data accordingly – clinical response and clinical remission (CDAI scores) at relevant intervals.
Other bias	Low risk	Baseline characteristics reported and balanced for participants in all groups. No other apparent sources of bias.

**Table:** Sands 2014

***Study characteristics***		
Methods	**Study design:** 2 arm, double‐blind randomised trial **Number of centres:** multicentre **Countries:** multiple across North America, Europe, Asia, Africa and Australia **Study dates:** November 2010 to April 2012 **Setting:** NR	
Participants	**Induction or maintenance study:** induction study **Active or inactive disease at beginning of study:** active Participants were considered for the primary outcome if they had failed TNF antagonists. They were also included for randomisation and some of the secondary outcomes if they were TNF antagonist naive. **Inclusion criteria** Aged 18–80 years CD with known ileal or colon (or both) involvement ≥ 3 months before enrolment (based on clinical and endoscopic evidence, corroborated by histopathology) At least moderately active CD (defined by CDAI score 220–400 within 7 days before enrolment) in addition to ≥ 1 of: CRP > 2.87 mg/L, colonoscopy within prior 4 months, fecal calprotectin > 250 μg/g during screening in conjunction with features of active CD on small bowel imaging Inadequate response, loss of response or intolerance to: TNF inhibitors, immunosuppressive or corticosteroids in past 5 years (for primary outcome population) **Exclusion criteria** Previous exposure to: vedolizumab, natalizumab, efalizumab or rituximab Concurrent lactation or pregnancy Unstable or uncontrolled medical condition Major neurological disorder Prior malignancies (except cancers where recurrence risk after adequate treatment was expected to be low) Active drug or alcohol dependence Active psychiatric disease or other complicating factors that could result in non‐adherence to study procedures **Baseline disease characteristics** CG = placebo IG = IV vedolizumab weeks 0, 2 and 6 Females: CG 57%, IG 56% Median age (years): CG 34.8 (IQR 19–77), IG 36.9 (IQR 20–69) Mean weight (kg): CG 71.3 (IQR 41–147), IG 69.5 (IQR 40–144) Median BMI (kg/m^2^): CG 23.3 (IQR 15–48), IG 23.3 (IQR 15–43) Mean CDAI: CG 301.3 (SD 55.0), IG 313.9 (SD 53.2) Mean CRP (mg/L): CG 18.5 (SD 22), IG 19.0 (SD 23.2) Mean fecal calprotectin (μg/g): CG 1426.5 (SD 2357.8), IG 1148.1 (SD 1878.6) Disease localisation – ileum only: CG 14%, IG 16% Disease localisation – colon only: CG 25%, IG 23% Disease localisation – both ileum and colon: CG 61%, IG 61% History of CD surgery: CG 43%, IG 44% History of fistulising disease: CG 37%, IG 34% Corticosteroid use: CG 52%, IG 53% Immunosuppressive use: CG 33%, IG 34% Mesalamine use: CG 29%, IG 33% Prior immunosuppressive exposure: CG 93%, IG 84% Prior TNF failure: CG 76%, IG 76%	
Interventions	**CG:** IV placebo at weeks 0, 2 and 6 **IG:** IV vedolizumab 300 mg at weeks 0, 2 and 6 **Duration of study:** 10 weeks **Measurement timepoints during study:** weeks 6 and 10 **Follow‐up measurements after study end:** those who had no unacceptable adverse events or did not require CD‐related surgery were continued in the open‐label GEMINI long‐term safety trial	
Outcomes	**Primary outcomes as defined by study authors** Proportion of participants in clinical remission at week 6 (defined by CDAI ≤ 150) from the TNF antagonist failure population **Secondary outcomes** Proportion of participants in clinical remission at week 6 from the overall study population (including about 25% of TNF antagonist‐naive participants) Proportion of participants in clinical remission at week 10 (from the TNF antagonist‐failure populations in addition to the additional TNF‐naive population) Proportion of participants with a CDAI‐100 response at week 6 (from the TNF antagonist‐failure population)	
Notes	**Funding source:** Takeda Pharmaceuticals International, Inc. Sponsored and funded by Millennium Pharmaceuticals, Inc (trading as Takeda Pharmaceuticals International Co). **Conflicts of interest:** (quote) "The authors disclose the following: Bruce Sands has received consulting and advisory board fees as well as clinical research/institutional grant support from AbbVie, Inc, Janssen Pharmaceuticals, Inc, and Takeda Pharmaceuticals International Co; Brian Feagan has received consulting fees and research grant support from Janssen Pharmaceuticals, Inc, Takeda Pharmaceuticals International Co, and UCB SA; Paul Rutgeerts has received consulting fees from Takeda Pharmaceuticals International Co. and UCB SA; Jean‐Frédéric Colombel has received consulting fees from Takeda Pharmaceuticals International Co. and UCB SA; William Sandborn has received consulting fees and research grants from Janssen Pharmaceuticals, Inc, Takeda Pharmaceuticals International Co, and UCB SA, and speaker fees from Janssen Pharmaceuticals, Inc; Richmond Sy has received consulting, lecture, and advisory board fees as well as research grant support from AbbVie, Inc, and Janssen Pharmaceuticals, Inc, and both advisory board fees and clinical trial support from Takeda Pharmaceuticals International Co; Geert D'Haens has received consulting and lecture fees from AbbVie, Inc, Janssen Pharmaceuticals, Inc, Takeda Pharmaceuticals International Co, and UCB SA, research grants from Janssen Pharmaceuticals, Inc, and speaking honoraria from UCB SA; Shomron Ben‐Horin has received consultancy and advisory board fees from Janssen Pharmaceuticals, Inc, and AbbVie, Inc, and an unrestricted research grant from Janssen Pharmaceuticals, Inc; Asit Parikh is an employee of Takeda Pharmaceuticals International, Inc; Jing Xu, Maria Rosario, Irving Fox, and Catherine Milch are employees of Takeda Pharmaceuticals International Co; and Stephen Hanauer has received consultancy and advisory board fees as well as clinical research/institutional grant support from AbbVie, Inc, Janssen Pharmaceuticals, Inc, Takeda Pharmaceuticals International Co, and UCB SA.	
***Risk of bias***		
**Bias**	**Authors' judgement**	**Support for judgement**
Random sequence generation (selection bias)	Low risk	Quote: "randomization was computer‐generated centrally."
Allocation concealment (selection bias)	Low risk	Quote: "patient enrollment, monitored by an interactive voice response system."
Blinding of participants and personnel (performance bias) All outcomes	Low risk	Quote: "treatment‐qualified patient received a unique randomization number used to provide treatment assignments for dose preparation via the interactive voice response system. Saline bag covers and labels maintained blinding. Only the study site pharmacist was aware of treatment assignments."
Blinding of outcome assessment (detection bias) All outcomes	Low risk	Contacted study authors by e‐mail (Dr Bruce Sands), who confirmed that outcome assessors were blinded to treatment assignments.
Incomplete outcome data (attrition bias) All outcomes	Low risk	Attrition in each group reported and balanced in all groups. Discontinuation due to adverse events reported and balanced between treatment and placebo groups.
Selective reporting (reporting bias)	Low risk	Although published protocol was reported to be available, we could not find it. According to trial registration and method section author reported relevant outcomes – proportion of participants in clinical remission and response (CDAI scores).
Other bias	Low risk	Baseline characteristics reported for and balanced in all groups. No other apparent sources of bias.

**Table:** Vermeire 2021

***Study characteristics***		
Methods	**Study design:** 2‐arm, randomised, double‐blind, placebo‐controlled, phase 3 trial **Number of centres:** multicentre (169) **Countries:** multiple (30) **Study dates:** December 2015 to May 2019 **Setting:** NR	
Participants	**Induction or maintenance study:** maintenance study **Active or inactive disease at beginning of study:** active disease although at commencement of randomisation were in remission. After a 28‐day screening period, all enroled participants received open‐label IV vedolizumab 300 mg at weeks 0 and 2 with disease assessment at week 6. **Those who responded** (CDAI ≥ 70 decrease in CDAI) were randomised 2:1 to maintenance vedolizumab **Inclusion criteria** Aged 18–80 years Diagnosis of CD established ≥ 3 months before screening by clinical and endoscopic evidence and corroborated by a histopathology report Moderate‐to‐severe active CD (CDAI score 220–450) within 7 days before the first dose of study drug and ≥ 1 of: CRP > 2.87 mg/L; ileocolonoscopy with a minimum of 3 non‐anastomotic ulcer or 10 aphthous ulcers, within 4 months before screening; or fecal calprotectin > 250 μg/g with CT/MRI/small bowel radiography/capsule endoscopy revealing CD ulcerations within 4 months before screening Ileal or colonic (or both) involvement at minimum People with > 8 years' duration of extensive colitis or pancolitis or left‐sided colitis of > 12 years' duration must have documented surveillance endoscopy performed within 12 months of screening Up‐to‐date cancer screening Inadequate response to, loss of response to or intolerance of ≥ 1 of: immunomodulators, corticosteroids or anti‐TNF therapies **Exclusion criteria** Gastrointestinal exclusion criteria Abdominal abscess, extensive colonic resection, subtotal or total colectomy History of > 3 small bowel resections or diagnosis of short bowel syndrome Received tube feeding, defined formula diets or parenteral alimentation within 28 days before the administration of the first dose of study drug Previous ileostomy, colostomy or known fixed symptomatic stenosis of the intestine Receipt of any investigational or approved biological/biosimilar within 60 days or 5 half‐lives of screening, or receipt of any non‐permitted investigational or approved non‐biological therapies within 30 days or 5 half‐lives of screening Oral 5‐ASA probiotics and antibiotics were permitted if doses were stable for 2 weeks before first dose of study and remained stable throughout the study. Antidiarrhoeals were permitted. Azathioprine, 6‐mercaptopurine or methotrexate could be continued if the participant's dose had been stable for 8 weeks before the start of the study Topical (rectal) treatment with 5‐ASAs or corticosteroid enemas/suppositories within 2 weeks of the administration of the first dose of study drug Requirement or anticipated requirement for surgical intervention for CD during the study History or evidence of adenomatous colonic polyps that had not been removed or colonic mucosal dysplasia Suspected or confirmed diagnosis of ulcerative colitis, indeterminate colitis, ischaemic colitis, radiation colitis, diverticular disease associated with colitis or microscopic colitis Infectious disease exclusion criteria Evidence of an active infection during the screening period Evidence of, or treatment for, *Clostridium difficile* infection or other intestinal pathogen within 28 days before the first dose of study drug People with chronic HBV infection or chronic HCV infection or HBV‐immune may have been included Active or latent tuberculosis Any identified congenital or acquired immunodeficiency (e.g. common variable immunodeficiency, HIV infection, organ transplantation) Receipt of any live vaccinations within 30 days before screening Clinically significant infection (e.g. pneumonia, pyelonephritis) within 30 days before screening, or ongoing chronic infection General exclusion criteria Previous exposure to approved or investigational anti‐integrin antibodies (e.g. natalizumab, efalizumab, etrolizumab, abrilumab (AMG 181)), antimucosal addressin cell adhesion molecule‐1 antibodies or rituximab Previous exposure to vedolizumab Hypersensitivity or allergies to any of the vedolizumab excipients Any unstable or uncontrolled cardiovascular, pulmonary, hepatic, renal, gastrointestinal, genitourinary, haematological, coagulation, immunological, endocrine/metabolic or other medical disorder that, in the opinion of the investigator, would confound the study results or compromise patient safety Any surgical procedure requiring general anaesthesia within 30 days before screening or plan to undergo major surgery during the study period Any history of malignancy, except for the following: adequately treated non‐metastatic basal cell skin cancer; squamous cell skin cancer that had been adequately treated and that had not recurred for ≥ 1 year before screening and history of cervical carcinoma in situ that had been adequately treated and that had not recurred for ≥ 3 years before screening. People with remote history of malignancy (e.g. > 10 years since completion of curative therapy without recurrence) were to be considered on a case‐by‐case basis based on the nature of the malignancy and the therapy received History of any major neurological disorders, including stroke, multiple sclerosis, brain tumour or neurodegenerative disease Positive PML subjective symptom checklist at screening (or before the administration of the first dose of study drug at week 0) Any of the following laboratory abnormalities during the screening period: haemoglobin level < 8 g/dL, white blood cell count < 3 × 10^9^/L, lymphocyte count < 0.5 × 10^9^/L, platelet count < 100 × 10^9^/L or > 1200 × 10^9^/L, alanine aminotransferase or aspartate aminotransferase > 3 × ULN, alkaline phosphatase > 3 × ULN, serum creatinine > 2 × ULN History of drug abuse (defined as any illicit drug use) or a history of alcohol abuse within 1 year before screening Active psychiatric problem that, in the investigator's opinion, may have interfered with compliance with study procedures Patient or carer was unable to attend all study visits or comply with study procedures Unwilling or unable to self‐inject, or did not have a carer (defined as a legal adult) to inject the study medication Lactation or pregnancy during the screening period or a positive urine pregnancy test at week 0, before study drug administration Intention to reproduce before, during or within 18 weeks after participating in study Immediate family member, study site employee or in a dependent relationship with a study site employee who was involved in conduct of study (e.g. spouse, parent, child, sibling), or may have consented under duress **Baseline disease characteristics** Mean age (years): CG 36.1 (SD 12.9), IG 38.2 (SD 13.9) Male: CG 49.3%, IG 57.1% White: CG 92.5%, IG 90.9% Mean weight (kg): CG 69.8 (SD 18.1), IG 74.1 (SD 19.0) Smoker: CG 19.4%, IG 19.6% Mean duration of CD (years): CG 8.2 (SD 8.4), IG 9.5 (SD 8.3) Moderate disease activity – CDAI ≤ 330: CG 60.4%, IG 58.2% Severe disease activity – CDAI > 330: CG 39.6%, IG 41.8% Median CDAI score at baseline: CG 309 (IQR 198–461), IG 318 (IQR 206–559) Median CDAI at week 6: CG 147.5 (IQR −3 to 326), IG 150.5 (IQR −8 to 362) Median fecal calprotectin (μg/g): CG 870.5 (range 10–15,050), IG 736 (range 10–14,570) CRP ≤ 2.87 mg/L: CG 23.9%, IG 26.2% CRP > 2.87 mg/L to ≤ 5 mg/L: CG 16.4%, IG 12.7% CRP > 5 mg/L to ≤ 10 mg/L: CG 15.7%, IG 23.6% CRP > 10 mg/L: CG 44%, IG 37.5% Disease location – ileum only: CG 15.7%, IG 24% Disease location – colon only: CG 19.4%, IG 20% Disease location – ileocolonic: CG 55.2%, IG 44.4% Disease location – other: CG 9.7%, IG 11.3% Prior surgery for CD: CG 25.4%, IG 27.6% Anti‐TNF naive: CG 47.8%, IG 40% Prior anti‐TNF use: CG 53%, IG 61.1% Prior use of immunomodulators only: CG 3%, IG 5.8% Prior use of oral corticosteroids only: CG 17.2%, IG 24.4% Prior use of oral corticosteroids and immunomodulator: CG 76.9%, IG 68.7% Concomitant medications – only immunomodulator: CG 25.4%, IG 18.5% Concomitant medications – only corticosteroids: CG 23.1%, IG 23.3% Concomitant medications – immunomodulators and corticosteroids: CG 9.7%, IG 11.3% History of fistulising disease: CG 25.4%, IG 19.3% Draining fistula at baseline: CG 9%, IG 5.1% Extraintestinal manifestations: CG 62.7%, IG 57.1%	
Interventions	**IG:** vedolizumab 108 mg subcutaneously every 2 weeks **CG:** placebo subcutaneously every 2 weeks **Duration of study:** 52 weeks **Measurement timepoints during study** Participants completed validated instruments to measure quality of life and work productivity at weeks 0, 6, 30 and 52, including the IBDQ Blood samples were drawn for determination of vedolizumab serum concentrations predose at weeks 0, 6, 8, 14, 22, 30, 38, 46, 50 and 68 **Any follow‐up measurements after study end?** NR	
Outcomes	**Primary outcomes as defined by study authors** Clinical remission (defined as CDAI score ≤ 150) at week 52 **Secondary outcomes as defined by study authors** Enhanced clinical response (defined as a ≥ 100 decline in CDAI score from baseline (week 0)) at week 52 Corticosteroid‐free clinical remission (participants using oral corticosteroid at baseline who discontinued corticosteroid and were in clinical remission at week 52) Clinical remission at week 52 in anti‐TNF‐naive participants	
Notes	**Funding source:** supported by Takeda. WJS was supported in part by the NIDDK‐funded San Diego Digestive Diseases Research Center (P30 DK120515). Medical writing support was provided by Rezan Sahinkaya, PhD, of ProEd Communications, Inc, and Milena Wagner, PhD, of Excel Medical Affairs, funded by Takeda. **Conflicts of interest:** (quote) "SV has received research support from AbbVie, Johnson & Johnson, Pfizer, and Takeda; lecture fees from AbbVie, Centocor, Ferring, Genentech/Roche, Hospira, Johnson & Johnson, Merck Sharp & Dohme, Pfizer, Takeda, and Tillotts; and consulting fees from AbbVie, Abivax, Celgene, Celltrion, Centocor, Ferring, Galapagos, Genentech/Roche, Gilead, GlaxoSmithKline, Hospira, Johnson & Johnson, Merck Sharp & Dohme, Mundipharma, Pfizer, ProDigest, Prometheus, Second Genome, Takeda, and Tillotts. GDH has received research support from AbbVie, Bühlmann Laboratories, Dr Falk Pharma, Ferring, GlaxoSmithKline, Johnson & Johnson, Medtronics, Merck Sharp & Dohme, Millennium/Takeda, PhotoPill, Prometheus, Robarts Clinical Trials, and Setpoint; lecture fees from AbbVie, Biogen, Dr Falk Pharma, Ferring, Giuliani, Johnson & Johnson, Merck Sharp & Dohme, Millennium/Takeda, Norgine, Otsuka, Shire, Tillotts, UCB, and Vifor; consulting fees from AbbVie, Ablynx, ActoGeniX, Amakem, Amgen, AM‐Pharma, AstraZeneca, Biogen, Boehringer Ingelheim, Bristol Myers Squibb, Celgene/Receptos, Celltrion, Cosmo, Elan, enGene, Ferring, Galapagos, Gilead, GlaxoSmithKline, Hospira/Pfizer, Immunic, Johnson & Johnson, Lycera, Medimetrics, Medtronics, Merck Sharp & Dohme, Millennium/Takeda, Mitsubishi Pharma, Mundipharma, Novo Nordisk, Otsuka, PDL, Prometheus, Protagonist, Salix, Samsung Bioepis, Sandoz, Setpoint, Shire, Teva, TiGenix, Tillotts, TopiVert, UCB, Versant, and Vifor; directorship at Robarts Clinical Trials. FB has received grant/research support from AbbVie, Amgen, Janssen, and Takeda; honoraria from AbbVie, Amgen, Arena, Celgene, Ferring, Fresenius Kabi, Janssen, Merck Sharp & Dohme, Pfizer, and Takeda; and was on speaker bureaus for AbbVie, Ferring, Janssen, Merck Sharp & Dohme, Pfizer, and Takeda. SD has received lecture and consulting fees from AbbVie, Allergan, Biogen, Boehringer Ingelheim, Celgene, Celltrion, Ferring, Hospira, Johnson & Johnson, Merck, Merck Sharp & Dohme, Mundipharma, Pfizer, Sandoz, Takeda, TiGenix, UCB, and Vifor. TK has received consulting fees from AbbVie, Alfresa, CovidienÐ, Eli Lilly, Ferring, Janssen, Kyorin, Mochida, Nippon Kayaku, Pfizer, Takeda, and Thermo Fisher Scientific; lecture fees from AbbVie, Ajinomoto Bio‐Pharma, Alfresa, Asahi Kasei Medical, Astellas, Celltrion, EA Pharma, Eisai, Gilead, Janssen, JIMRO, Kyorin, Mitsubishi Tanabe Pharma, Mochida, Nippon Kayaku, Takeda, and Zeria; and research support from AbbVie, Alfresa, Asahi Kasei Medical, EA Pharma, Kyorin, Mochida, Nippon Kayaku, Otsuka, Thermo Fisher Scientific, and Zeria. EVL Jr has received research support from AbbVie, Amgen, Bristol Myers Squibb, Celgene, Genentech, Gilead, Janssen, Pfizer, Receptos, Robarts Clinical Trials, Takeda, Theravance Biopharma, and UCB; consulting fees from AbbVie, Allergan, Amgen, Arena, Boehringer Ingelheim, Bristol Myers Squibb, Calibr, Celgene, Celltrion, Eli Lilly, Genentech, Gilead, Iterative Scopes, Janssen, Ono Pharma, Pfizer, Sun Pharma, Takeda, and UCB; is a shareholder of Exact Sciences; and served as a medical editor for Healio/SLACK. SB, WZ, and KK are employees of Millennium Pharmaceuticals, a Takeda group company and hold stock/stock options in Takeda. CA is an employee of Takeda and holds stock/stock options in Takeda. MR and CC were employees of Millennium Pharmaceuticals, a Takeda group company at the time this research was conducted. WJS has received research grants from AbbVie, Abivax, Arena Pharmaceuticals, Boehringer Ingelheim, Celgene, Genentech, Gilead Sciences, GlaxoSmithKline, Janssen, Lilly, Pfizer, Prometheus Biosciences, Seres Therapeutics, Shire, Takeda, and Theravance Biopharma; consulting fees from AbbVie, Abivax, AdMIRx, Alfasigma, Alimentiv [previously Robarts Clinical Trials, owned by Alimentiv Health Trust], Alivio Therapeutics, Allakos, Amgen, Applied Molecular Transport, Arena Pharmaceuticals, Bausch Health [Salix], Beigene, Bellatrix Pharmaceuticals, Boehringer Ingelheim, Boston Pharmaceuticals, Bristol Myers Squibb, Celgene, Celltrion, Cellularity, Cosmo Pharmaceuticals, Escalier Biosciences, Equillium, Forbion, Genentech/Roche, Gilead Sciences, Glenmark Pharmaceuticals, Gossamer Bio, Immunic [Vital Therapies], Index Pharmaceuticals, Intact Therapeutics, Janssen, Kyverna Therapeutics, Landos Biopharma, Lilly, Oppilan Pharma, Otsuka, Pandion Therapeutics, Pfizer, Progenity, Prometheus Biosciences, Protagonists Therapeutics, Provention Bio, Reistone Biopharma, Seres Therapeutics, Shanghai Pharma Biotherapeutics, Shire, Shoreline Biosciences, Sublimity Therapeutics, Surrozen, Takeda, Theravance Biopharma, Thetis Pharmaceuticals, Tillotts Pharma, UCB, Vendata Biosciences, Ventyx Biosciences, Vimalan Biosciences, Vivelix Pharmaceuticals, Vivreon Biosciences, and Zealand Pharma; and holds stock/stock options in Allakos, BeiGene, Gossamer Bio, Oppilan Pharma, Prometheus Biosciences, Progenity, Shoreline Biosciences, Ventyx Biosciences, Vimalan Biosciences, and Vivreon Biosciences; spouse of WJS reports consulting for Iveric Bio and Oppilan Pharma; and holds stock/stock options in Iveric Bio, Oppilan Pharma, Progenity, Prometheus Biosciences, Ventyx Biosciences, and Vimalan Biosciences; employee of Prometheus Biosciences."	
***Risk of bias***		
**Bias**	**Authors' judgement**	**Support for judgement**
Random sequence generation (selection bias)	Low risk	Quote from the legend of Supplementary figure 2, "randomisation personnel of the sponsor or designee generated the randomisation schedule before the start of the study. An interactive web response system was used for patient randomisation." We contacted the authors who confirmed the randomisation schedules were generated using interactive response technology.
Allocation concealment (selection bias)	Low risk	Quote: "An interactive web response system was used for patient randomisation" and "All randomisation information was stored in a secured area, accessible only by authorised personnel." We contacted the authors who confirmed that the personnel from the vendor who had access to the randomisation schedule were not involved in the study conduct or data analysis.
Blinding of participants and personnel (performance bias) All outcomes	Low risk	Quote: "double‐blind, placebo‐controlled" and "All randomisation information was stored in a secured area, accessible only by authorised personnel." We contacted the authors who confirmed participants and study personnel were unaware of treatment assignments.
Blinding of outcome assessment (detection bias) All outcomes	Low risk	We contacted the authors (Dr Severine Vermeire), who confirmed that outcome assessors were blinded to the treatment assignments.
Incomplete outcome data (attrition bias) All outcomes	Low risk	Attrition was accounted for and balanced in both groups with adequate reasons provided for loss in numbers.
Selective reporting (reporting bias)	Low risk	No published protocol found. According to trial registration and method section, authors reported the necessary endpoints – proportion of participants with clinical remission and response.
Other bias	Low risk	Baseline characteristics were reported for and balanced in all groups. No other apparent sources of bias.

**Table:** Watanabe 2020 – Induction Phase

***Study characteristics***		
Methods	**Study design:** 2‐arm, phase 3, double‐blind randomised study **Number of centres:** multicentre (77 centres) **Countries:** Japan **Study dates:** January 2014 to November 2017 **Setting:** NR	
Participants	**Induction or maintenance study:** induction (maintenance trial assessed separately) **Active or inactive disease at beginning of study:** active disease **Inclusion criteria** In opinion of investigator, the person was capable of understanding and complying with protocol requirements Person or, when applicable, the person's legally acceptable representative signed and dated the informed consent form prior to initiation of any study procedures Male or female, aged 15–80 years, inclusive, at signing of informed consent A non‐sterilised male participant who had a female partner of child‐bearing potential had to agree to use adequate contraception during the period from the signing of informed consent to 6 months after the last dose of the study drug A female participant of child‐bearing potential (i.e. non‐sterilised or whose last regular menses was within previous 2 years) who had a non‐sterilised male partner had to agree to use adequate contraception during the period from the signing of informed consent to 6 months after the last dose of the study drug Diagnosed with ileal, colonic or ileocolonic CD ≥ 3 months prior to first dose of study drug according to the Revised Diagnostic Criteria for CD issued by Research Group for Intractable Inflammatory Bowel Disease Designated by the Ministry of Health, Labor, and Welfare of Japan (2012) CDAI score 220–450 (inclusive) at first dose of study drug, and meeting ≥ 1 of: CRP > 0.30 mg/dL at screening; irregular‐to‐round shaped ulcers or multiple aphtha (≥ 10 lesions) in extensive area of the small or large intestine on endoscopy or imaging test within 4 months before first dose of study drug; longitudinal ulcers or a cobblestone appearance in the small or large intestine on endoscopy or imaging test within 4 months before first dose of study drug Complication of colon cancer or dysplasia was ruled out by total colonoscopy at first dose of study drug (or the results from total colonoscopy performed within 1 year before giving consent were available), if patients met any of: extensive or limited colitis of ≥ 8 years' duration, aged ≥ 50 years or with a first‐degree family history of colon cancer Met the treatment failure criteria below with ≥ 1 of the following agents within 5 years before signing of informed consent Corticosteroids Resistance: patients whose response was inadequate despite the treatment of ≥ 40 mg/day (oral or IV) for ≥ 1 week or 30–40 mg/day (oral or IV) for ≥ 2 weeks Dependence: patients who had failed to reduce the dosage to < 10 mg/day due to recurrence during gradual dose reduction (oral or IV) Intolerance: patients who were unable to receive continuous treatment due to adverse reactions (e.g. Cushing's syndrome, osteopenia/osteoporosis, hyperglycaemia, insomnia, infection) Immunomodulators (azathioprine, 6‐mercaptopurine or methotrexate) Refractory: patients whose response was inadequate despite the treatment for ≥ 12 weeks Intolerance: patients who were unable to receive continuous treatment due to adverse reactions (e.g. nausea/vomiting, abdominal pain, pancreatitis, liver function test abnormalities, lymphopenia, thiopurine S‐methyltransferase genetic mutation, infection) Anti‐TNFα Inadequate response: patients whose response was considered inadequate (determined by investigators) despite the induction therapy in the dosage described in the package insert (this definition was different from the 1 used in GEMINI 2 and GEMINI 3) Loss of response: patients who had relapse during the scheduled maintenance therapy after achieving clinical response (those who withdrew for other reasons than relapse were not applicable here) Intolerance: patients who were unable to receive continuous treatment due to adverse reactions (e.g. infusion‐related reaction, demyelinating disease, congestive heart failure, infection) **Exclusion criteria** Evidence of or suspected abscess History of subtotal or total colectomy History of small intestine resections in ≥ 3 locations, or a history of diagnosis of short bowel syndrome Had ileostomy, colostomy, internal fistula, or severe intestinal stenosis Treatment history with natalizumab, efalizumab or rituximab Started oral 5‐ASAs, probiotics, antibiotics for CD treatment or oral corticosteroids (≤ 30 mg/day) within 13 days before first dose of study drug. Or patients who changed dosage of or discontinued these drugs within 13 days before first dose of study drug if the patient had used these drugs for > 14 days before first dose of study drug Received 5‐ASA, corticosteroid enemas/suppositories, corticosteroid IV infusion, oral corticosteroid at > 30 mg/day, drugs for diarrhoea‐predominant irritable bowel syndrome, or Chinese herbal medicine for CD treatment (e.g. Daikenchuto) within 13 days before first dose of study drug Received azathioprine, 6‐mercaptopurine or methotrexate within 27 days before first dose of study drug. However, this did not apply to patients who had used these drugs for > 83 days before first dose of study drug and continued the steady dose of the drugs for > 27 days before first dose of study drug Received ciclosporin, tacrolimus, tofacitinib or any study drugs of low molecular compound for CD treatment within 27 days before first dose of study drug Received adalimumab within 27 days before first dose of study drug or any biologicals other than adalimumab within 55 days before first dose of study drug. However, this did not apply to patients who had received localised injections of these drugs (e.g. intraocular injection for treatment of age‐related macular degeneration) Received any live‐vaccinations within 27 days before first dose of study drug Undergone an enterectomy within 27 days before the first dose of study drug or those who anticipated an enterectomy during study Received leukocytapheresis or granulocyte apheresis within 27 days before first dose of study drug Received central venous nutrition therapy or enteral total nutrition therapy, or fasted within 20 days before first dose of study drug Received an enteral nutrient of > 900 kcal/day or who had started an enteral nutrient of ≤ 900 kcal/day within 20 days before first dose of study drug. Or patients who changed dosage of or discontinued the enteral nutrient within 20 days before first dose of study drug if the patient had received an enteral nutrient of ≤ 900 kcal/day > 21 days before first dose of study drug Infected with *Clostridium difficile*, cytomegalovirus or any other intestinal pathogen within 27 days before first dose of study drug Evidence of adenomatous colonic polyps that needed to be removed at first dose of study drug History or a complication of large or small intestinal dysplasia Suspected to have enteritis other than CD Hepatitis B surface (HBs) antigen‐positive or HCV antibody‐positive at the screening. Or patients who were hepatitis B core (HBc) antibody‐positive or HBs antibody‐positive, even though HBs antigen‐negative. However, this did not apply to patients who were only HBs antibody‐positive due to HBV vaccination, HBV‐DNA‐negative, HCV antigen‐negative or HCV‐RNA‐negative Evidence of history of tuberculosis or a suspected history of tuberculosis (including those who had findings suggesting previous tuberculosis on chest imaging procedure at the screening). However, this did not apply to patients who had completed prophylactic isoniazid, or patients who had been receiving prophylactic isoniazid for > 21 days before first dose of study drug (in the latter case, the screening period was allowed to extend up to 28 days to ensure ≥ 21‐day prophylactic isoniazid and then the study treatment was allowed to start) Positive T‐SPOT test or QuantiFERON test at screening History or complication of identified congenital or acquired immunodeficiency syndrome (e.g. not‐classifiable immunodeficiency, HIV infection or organ transplantation) Affected by extraintestinal infection (e.g. pneumonia, sepsis, active hepatitis or pyelonephritis) within 27 days before first dose of study drug Treatment history with vedolizumab (MLN0002) Females who were lactating at screening, or had positive urine pregnancy test either at screening or baseline Serious complications in the heart, lung, liver, kidney, metabolism, gastrointestinal system, urinary system, endocrine system or blood History of a surgery requiring general anaesthesia within 27 days before first dose of study drug, or with a schedule of a surgery requiring hospitalisation during study period Complication or a history of malignancy. However, this did not apply to the following: patients who had a curative resection of localised skin basal cell carcinoma or had completed curative radiotherapy patients who had not experienced recurrence for > 1 year since completion of a curative resection or curative radiotherapy for skin squamous cell carcinoma patients who had not experienced recurrence for > 3 years since completion of a curative resection or curative radiotherapy for intraepithelial carcinoma of uterine cervix For patients who had a substantially distant history of malignancy (e.g. ≥ 10 years without recurrence since treatment completion), the investigator and the sponsor were to have a discussion to decide eligibility on the basis of type of malignancy and treatment applied History or a complication of the central nervous disorder, including stroke, multiple sclerosis, brain tumour or neurodegenerative disease Any subjective symptoms in the subjective PML checklist at the screening or baseline Any of the following laboratory abnormalities at the screening; haemoglobin ≤ 8 g/dL, white blood cell count ≤ 3000/μL, lymphocytes ≤ 500/μL, platelets ≤ 100,000/μL or ≥ 1,200,000/μL, alanine aminotransferase or aspartate aminotransferase ≥ 3 × ULN, alkaline phosphatase ≥ 3 × ULN, creatinine ≥ 2 × ULN History or a complication of alcohol dependence or illicit drug use within 1 year before first dose of study drug History or a complication of psychotic disorder that could obstruct compliance with study procedures **Baseline disease characteristics** CG: IV placebo at weeks 0, 2 and 6 IG: IV vedolizumab 300 mg weeks 0, 2 and 6 Mean age (years): CG 32.6 (SD 10.9), IG 33.9 (SD 12.3) Male: CG 66.7%, IG 64.6% Mean BMI (kg/m^2^): CG 19.8, IG 21.2 Mean duration of CD (years): CG 9.1 (SD 6.5), IG 9 (SD 6.2) Mean CRP (mg/L): CG 2.9 (SD 3.2), IG 2.2 (SD 2.2) CDAI at week 0: ≤ 220: CG 6.4%, IG 0% CDAI at week 0: > 220 to ≤ 330: CG 64.1%, IG 70.9% CDAI at week 0: > 330 to ≤ 450: CG 26.9%, IG 25.3% CDAI at week 0: > 450: CG 2.6%, IG 3.8% Mean CDAI at week 0: CG 295 (SD 64.8), IG 303.9 (SD 63.2) Location of the lesion – ileal: CG 11.5%, IG 16.5% Location of the lesion – colonic: IG 24.4%, CG 13.9% Location of the lesion – ileocolonic: IG 64.1%, CG 63.2% Never‐smoker: CG 53.8%, IG 58.2% Current smoker: CG 14.1%, IG 16.5% Ex‐smoker: CG 32.1%, IG 25.3% Surgical history for CD: CG 38.5%, IG 30.4% Current medical condition related to fistula: CG 15.4%, IG 8.9% Prior anti‐TNF treatment: CG 79.5%, IG 77.2% Prior anti‐TNF failure – inadequate response: CG 12.8%, CG 17.7% Prior anti‐TNF failure – loss of response: CG 59%, IG 57% Prior anti‐TNF failure – intolerance: CG 6.4%, IG 1.3% Number of drugs of anti‐TNF failure – 0: CG 21.8%, IG 24.1% Number of drugs of anti‐TNF failure – 1: CG 37.2%, IG 36.7% Number of drugs of anti‐TNF failure – 2: CG 41%, IG 39.2% Prior immunomodulator failure: CG 51.3%, IG 49.4% Prior immunomodulator failure – refractory: CG 37.2%, IG 35.4% Prior immunomodulator failure – intolerance: CG 14.1%, IG 13.9% Prior corticosteroids failure: CG 32.1%, IG 27.8% Prior corticosteroids failure – resistance: CG 7.7%, IG 6.3% Prior corticosteroids failure – dependence: CG 16.7%, IG 21.5% Prior corticosteroids failure – intolerance: CG 7.7%, IG 0% Worst prior treatment failure – prior anti‐TNF failure: CG 78.2%, IG 75.9% Worst prior treatment failure – prior immunomodulator failure but not anti‐TNF failure: CG 11.5%, IG 15.2% Worst prior treatment failure – prior corticosteroid failure only: CG 10.3%, IG 8.9% Concomitant medications for CD at baseline – enteral nutrient: CG 55.1%, IG 48.1% Concomitant medications for CD at baseline – 5‐ASA: CG 75.6%, IG 81% Concomitant medications for CD at baseline – oral corticosteroids and no immunomodulator: CG 9%, IG 16.5% Concomitant medications for CD at baseline – no oral corticosteroids and no immunomodulator: CG 39.7%, IG 38% Concomitant medications for CD at baseline – oral corticosteroids and immunomodulator: CG 14.1%, IG 11.4%	
Interventions	**CG:** IV placebo at weeks 0, 2 and 6 **IG:** IV vedolizumab 300 mg weeks 0, 2 and 6 **Duration of study:** 14‐week induction phase **Measurement timepoints during study:** weeks 2, 6, 10 and 14 **Follow‐up measurements after study end:** participants were either included in the maintenance phase or in an open‐label cohort and reinduced with vedolizumab. Participants were followed up until week 94	
Outcomes	**Primary outcomes as defined by study authors** CDAI‐100 response at week 10 (CDAI reduction of ≥ 100) **Secondary outcomes** Percentage of participants who achieved clinical remission at week 10 (CDAI ≤ 150) Change over time in CRP concentration during the induction phase in participants with baseline CRP 0.30 mg/dL	
Notes	**Funding:** Takeda Pharmaceutical Company Limited.	
***Risk of bias***		
**Bias**	**Authors' judgement**	**Support for judgement**
Random sequence generation (selection bias)	Unclear risk	Quote: "Randomization schedules were generated by personnel designated by the sponsor." We contacted the authors to confirm what method was used to generate the schedule but received no response.
Allocation concealment (selection bias)	Low risk	Quote: "allocations were not disclosed until opening of the study drug allocation table, except to unblinded pharmacists at each site."
Blinding of participants and personnel (performance bias) All outcomes	Low risk	Quote: "allocations were not disclosed until opening of the study drug allocation table, except to unblinded pharmacists at each site."
Blinding of outcome assessment (detection bias) All outcomes	Unclear risk	Quote: "allocations were not disclosed until opening of the study drug allocation table, except to unblinded pharmacists at each site." We contacted the author by e‐mail (Dr Toshifumi Hibi) to confirm outcome assessor was unaware of the treatment assignments but received no response.
Incomplete outcome data (attrition bias) All outcomes	Low risk	Attrition was accounted for and balanced in all groups with adequate reasons provided for loss in numbers.
Selective reporting (reporting bias)	Low risk	No published protocol found. According to trial registration and method section, authors reported the relevant endpoints – proportion of participants with specific CDAI‐100 response and clinical remission.
Other bias	Low risk	Baseline characteristics were reported for and balanced in all groups. No other apparent sources of bias.

**Table:** Watanabe 2020 – Maintenance Phase

***Study characteristics***		
Methods	**Study design:** 2‐arm, double‐blind, randomised, controlled, parallel‐group study **Number of centres:** multicentre (77 centres) **Countries:** Japan **Study dates:** first participant screened 28 January 2014 and last participant visit was 16 November 2017 **Setting:** educational and research institutions, secondary care	
Participants	**Induction or maintenance study:** maintenance study (induction study assessed separately) **Active or inactive disease at beginning of study:** inactive disease*. NOTE – we treated responders (achieved CDAI reduction of 70 from baseline i.e. CDAI‐70) from the induction phase as having inactive CD for the maintenance part because data are scarce on maintenance efficacy in general. In reality, the participants would be a mixture of active (generally defined by CDAI > 150) and inactive (generally defined by CDAI < 150) CD. The proportion below and above CDAI 150 are not provided in the paper; therefore, numbers per disease status could not be provided. **Inclusion criteria** Patients needed to have achieved CDAI‐70 response at week 10 and to have completed the induction phase to be eligible for the maintenance phase of study. The inclusion criteria for the previous induction phase are given under Watanabe 2020 – Induction Phase. **Baseline disease characteristics** IG: vedolizumab 300 mg IV at week 14 then every 8 weeks until week 54 CG: placebo IV at week 14 then every 8 weeks until week 54 Mean age (years): CG 35.2 (SD 13), IG 36.7 (SD 16.8) Male: CG 75%, IG 50% Mean BMI (kg/m^2^): CG 21.9 (SD 3.7), IG 22.1 (SD 6.2) Mean duration of CD (years): CG 6.6 (SD 7.5), IG 9 (SD 4.9) Mean CRP (mg/L): CG 2.4 (SD 2.5), IG 2 (SD 1.6) CDAI at week 0: ≤ 220: CG 0%, IG 0% CDAI at week 0: > 220 to ≤ 330: CG 66.7%, IG 58.3% CDAI at week 0: > 330 to ≤ 450: CG 25%, IG 41.7% CDAI at week 0: > 450: CG 8.3%, IG 0% CDAI at week 10: ≤ 150: CG 50%, IG 66.7% CDAI at week 10: > 150 to ≤ 220: CG 33.3%, IG 8.3% CDAI at week 10: > 220 to ≤ 330: CG 16.7%, IG 25% Mean CDAI at week 0: CG 303.3 (SD 81.7), IG 319.8 (SD 79.3) Mean CDAI at week 10: CG 149.7 (SD 59.9), IG 147.9 (SD 89.2) Location of the lesion – ileal: CG 16.7%, IG 16.7% Location of the lesion – colonic: IG 8.3%, CG 41.7% Location of the lesion – ileocolonic: IG 75%, CG 41.7% Never‐smoker: CG 41.7%, IG 83.3% Current smoker: CG 8.3%, IG 0% Ex‐smoker: CG 50%, IG 16.7% Surgical history for CD: CG 25%, IG 25% Current medical condition related to fistula: CG 8.3%, IG 0% Prior anti‐TNF treatment: CG 58.3%, IG 66.7% Prior anti‐TNF failure – inadequate response: CG 8.3%, CG 25% Prior anti‐TNF failure – loss of response: CG 50%, IG 41.7% Prior anti‐TNF failure – intolerance: CG 0%, IG 0% Number of drugs of anti‐TNF failure – 0: CG 41.7%, IG 33.3% Number of drugs of anti‐TNF failure – 1: CG 41.7%, IG 16.7% Number of drugs of anti‐TNF failure – 2: CG 16.7%, IG 50% Prior immunomodulator failure: CG 50%, IG 58.3% Prior immunomodulator failure – refractory: CG 25%, IG 33.3% Prior immunomodulator failure – intolerance: CG 25%, IG 25% Prior corticosteroids failure: CG 33.3%, IG 33.3% Prior corticosteroids failure – resistance: CG 16.7%, IG 0% Prior corticosteroids failure – dependence: CG 16.7%, IG 33.3% Prior corticosteroids failure – intolerance: CG 0%, IG 0% Worst prior treatment failure – prior anti‐TNF failure: CG 58.3%, IG 66.7% Worst prior treatment failure – prior immunomodulator failure but not anti‐TNF failure: CG 16.7%, IG 25% Worst prior treatment failure – prior corticosteroid failure only: CG 25%, IG 8.3% Concomitant medications for CD at baseline – enteral nutrient: CG 41.7%, IG 66.7% Concomitant medications for CD at baseline – 5‐ASA: CG 91.7%, IG 66.7% Concomitant medications for CD at baseline – oral corticosteroids and no immunomodulator: CG 25%, IG 16.7% Concomitant medications for CD at baseline – no oral corticosteroids and no immunomodulator: CG 25%, IG 8.3% Concomitant medications for CD at baseline – oral corticosteroids and immunomodulator: CG 0%, IG 25%	
Interventions	**IG:** vedolizumab 300 mg IV at week 14 then every 8 weeks until week 54 **CG:** placebo IV at week 14 then every 8 weeks until week 54 (agent and dose NR) **Duration of study:** 46 weeks (week 14 to week 60) **Measurement timepoints during study:** blood samples collected immediately before administration at each visit, at weeks 22, 30 and 60 for the pharmacokinetic endpoint (serum vedolizumab concentration) and at weeks 30, 60 and 16 weeks after the last dose for the immunogenicity endpoint (appearance in serum of anti‐vedolizumab antibody, including neutralising antibody, determined by enzyme‐linked immunosorbent assay and electrochemoluminescent assay, respectively) **Follow‐up measurements after study end:** NR	
Outcomes	**Primary outcomes as defined by study authors** Percentage of participants who achieved clinical remission at week 60, as defined by CDAI ≤ 150 **Secondary outcomes as defined by study authors** Percentage of participants with CDAI‐100 response (CDAI reduction of ≥ 100 from baseline) at week 60 Percentage of participants with durable remission during the maintenance phase (defined as clinical response at ≥ 80% of the scheduled visits including week 60) Percentage of participants who received concomitant oral corticosteroids at baseline and showed clinical response at week 60 without corticosteroids (corticosteroid‐free remission).	
Notes	**Funding source:** Takeda Pharmaceutical Company Limited **Conflicts of interest:** Kenji Watanabe has received honoraria and research funding from AbbVie GK (AbbVie), Mitsubishi Tanabe Pharma (Mitsubishi Tanabe), EA Pharma (EA), Takeda Pharmaceutical (Takeda), Kyorin Pharmaceutical (Kyorin), Mochida Pharmaceutical (Mochida) and Janssen Pharmaceutical (Janssen); research funding from Astellas Pharma (Astellas), JIMRO, Zeria Pharmaceutical (Zeria), Otsuka Pharmaceutical (Otsuka) and Asahi Kasei Medical (Asahi Kasei). Satoshi Motoya has received honoraria and research funding from Janssen and Takeda; honoraria from Mitsubishi Tanabe, and Mochida; research funding from Pfizer Japan (Pfizer). Haruhiko Ogata has received honoraria from Takeda; research funding from Mitsubishi Tanabe, Mochida, Pfizer and AbbVie. Takanori Kanai has received honoraria and research funding from Mitsubishi Tanabe, Miyarisan Pharmaceutical (Miyarisan) and Takeda; honoraria from Astellas and AstraZeneca; research funding from EN Otsuka, Ezaki Glico, Otsuka, AbbVie, Mochida, Kyorin, Daiichi Sankyo, Nippon Kayaku, Yakult, Zeria, Sumitomo Dainippon Pharma, Ono Pharmaceutical, EA, Eisai, JIMRO, Chugai Pharmaceutical (Chugai) and UCB Japan (UCB). Toshiyuki Matsui has received honoraria and research funding from EA, Ajinomoto Seiyaku (Ajinomoto), AbbVie, Eisai, Kyorin, Zeria, Takeda, Mitsubishi Tanabe and Mochida; research funding from Miyarisan, Otsuka, Asahi Kasei, Astellas, AstraZeneca, MSD, JIMRO, Taiho Pharmaceutical (Taiho), Daiichi Sankyo, Nippon Kayaku, Kyowa Hakko Kirin, UCB and Chugai. Yasuo Suzuki has received honoraria and research funding from Mitsubishi Tanabe, AbbVie, EA and Mochida; honoraria from Janssen, Zeria and Kyorin; research funding from JIMRO, Kissei and Nippon Kayaku. Mitsuhiro Shikamura, Kenkichi Sugiura, Kazunori Oda, Tetsuharu Hori, and Takahiro Araki are employees of Takeda. Mamoru Watanabe has received honoraria and research funding from Mitsubishi Tanabe, Takeda, EA, Zeria and Gilead Sciences; honoraria from Ajinomoto, Janssen, Celltrion Healthcare and Pfizer; research funding from Nippon Kayaku, Mochida, Kissei, Miyarisan, Asahi Kasei, JIMRO, Kyorin, AbbVie, Kyowa Hakko Kirin, Kaken Pharmaceutical, Alfresa Pharma, Ayumi 304 J Gastroenterol (2020) 55:291–306 Pharmaceutical, Astellas, MSD, Daiichi Sankyo, Taiho, Toray Industries, Chugai and Fujirebio. Toshifumi Hibi has received honoraria and research funding from EA and Zeria; honoraria from Takeda, Mitsubishi Tanabe, AbbVie, Zeria, JIMRO, Janssen and Pfizer; research funding from Nippon Kayaku and Mochida.	
***Risk of bias***		
**Bias**	**Authors' judgement**	**Support for judgement**
Random sequence generation (selection bias)	Unclear risk	Quote: "Randomization schedules were generated by personnel designated by the sponsor." We contacted the authors to confirm what method they used to generate the schedule but received no response.
Allocation concealment (selection bias)	Low risk	Quote: "allocations were not disclosed until opening of the study drug allocation table, except to unblinded pharmacists at each site."
Blinding of participants and personnel (performance bias) All outcomes	Low risk	Quote: "allocations were not disclosed until opening of the study drug allocation table, except to unblinded pharmacists at each site."
Blinding of outcome assessment (detection bias) All outcomes	Unclear risk	Quote: "allocations were not disclosed until opening of the study drug allocation table, except to unblinded pharmacists at each site." We contacted the author by e‐mail (Dr Toshifumi Hibi) to confirm outcome assessor was unaware of the treatment assignments but received no response.
Incomplete outcome data (attrition bias) All outcomes	Low risk	Attrition was accounted for and balanced in all groups with adequate reasons provided for loss in numbers.
Selective reporting (reporting bias)	Low risk	No published protocol found. According to trial registration and method section authors reported the relevant endpoints – proportion of participants with specific CDAI‐100 response and clinical remission.
Other bias	Low risk	Baseline characteristics were reported for and balanced in all groups. No other apparent sources of bias.

5‐ASA: 5‐aminosalicylic acid; CDAI: Crohn's Disease Activity Index; CG: control group; CRP: C‐reactive protein; CT: computer tomography; HBV: hepatitis B virus; HCV: hepatitis C virus; IBDQ: Inflammatory Bowel Disease Questionnaire; IG: intervention group; IQR: interquartile range; IV: intravenous; NR: not reported; MRI: magnetic resonance imaging; PML: progressive multifocal leukoencephalopathy; SD: standard deviation; TNF: tumour necrosis factor; ULN: upper limit of normal.

### Characteristics of excluded studies [ordered by study ID]

**Table:**

Study	Reason for exclusion
Pipek 2020	Not a randomised trial
Sandborn 2014	Not a randomised trial
Vermeire 2017	Not a randomised trial

## Differences between protocol and review

We made the following changes from our protocol (Hui 2020).

**Methods – types of studies**

Quasi‐randomised trials were eligible in the final review, where they were not specifically referred to in the protocol.

**Methods – types of participants**

The types of participants were initially planned to include only a medically induced remission with exclusion of people with Crohn's‐related surgery in the preceding six months. We removed this requirement as these data were not available for any one of the included studies.

We clarified in the review that studies with only a subset of eligible participants would be included.

**Methods – types of interventions**

The protocol stated that vedolizumab was to be compared to a control arm of placebo or other medical therapy. We changed this to a comparison with placebo only in the review.

We provided more details on the intervention of interest (doses, frequency, duration) in the review.

**Methods – primary and secondary outcomes**

We reversed the primary outcomes from "failure to achieve clinical remission" to "induction of clinical remission" and "clinical relapse" to "maintenance of clinical remission".

We reversed the secondary outcomes from "failure to achieve clinical response" to "induction of clinical response", "failure to achieve endoscopic remission" to "endoscopic remission" and "failure to achieve endoscopic response" to "endoscopic response". The remaining secondary outcomes did not differ.

Primary and secondary outcomes were initially grouped in the protocol. In our review, we separated outcomes and studies into an induction phase (with primary and secondary outcomes) and maintenance phase (with primary and secondary outcomes) as several of the included studies conducted separate randomisation processes at the maintenance phase.

**Methods – search methods**

In our protocol, we planned to contact experts in the field for additional published and unpublished studies. We omitted this from the review. We removed the Cochrane IBD Review Group Specialised Trials Register as it is now integrated within CENTRAL.

For the search strategy for ClinicalTrials.gov, we included further details on the specific search. We included the WHO ICTRP search strategy.

In the search methods of the protocol, we stated that we would contact experts in the field. We removed this. However, we contacted the authors of the included studies. We also removed the manual search for conference abstracts because all relevant conferences publish the abstracts as a supplementary issue in journals and are indexed in Embase. We searched Embase including the conference abstracts.

**Methods – timing of outcome measurement**

The protocol stated assessments at six, 12 and 52 weeks. We replaced these timepoints with separate analyses for induction studies and maintenance studies. Timing of assessment of outcomes within induction and maintenance studies were as defined by the study authors.

**Methods – measures of treatment effect**

In the protocol, we described our plans to deal with continuous and time‐to‐event data. This was not required with the available studies.

**Methods – assessment of risk of bias in included studies**

We specified that we used the RoB 1 tool to determine the risk of bias of included studies.

**Methods – unit of analysis issues**

In the review, we have outlined how data from cluster randomised controlled trials would be incorporated.

**Methods – subgroup analyses**

We initially planned a subgroup analysis for participants on concomitant medications. However, the included studies did not report these.

**Methods – summary of findings**

We changed the plan for the summary of findings tables as not all outcomes were available. After peer review feedback, we removed subgroup analyses from the summary of findings tables. We also added the GRADE section of methods, description of our use of optimum information size to inform imprecision judgements and how we informed this.
